# Finite-Time Pinning Synchronization Control for T-S Fuzzy Discrete Complex Networks with Time-Varying Delays via Adaptive Event-Triggered Approach

**DOI:** 10.3390/e24050733

**Published:** 2022-05-21

**Authors:** Xiru Wu, Yuchong Zhang, Qingming Ai, Yaonan Wang

**Affiliations:** 1 School of Electronic Engineering and Automation, Guilin University of Electronic Technology, Guilin 541004, China; qmai@mails.guet.edu.cn; 2 School of Electrical and Information Engineering, Hunan University, Changsha 410114, China; yaonan@hnu.edu.cn

**Keywords:** discrete complex networks, T-S fuzzy model, pinning control, finite-time synchronization, adaptive event-triggered approach

## Abstract

This paper is concerned with the adaptive event-triggered finite-time pinning synchronization control problem for T-S fuzzy discrete complex networks (TSFDCNs) with time-varying delays. In order to accurately describe discrete dynamical behaviors, we build a general model of discrete complex networks via T-S fuzzy rules, which extends a continuous-time model in existing results. Based on an adaptive threshold and measurement errors, a discrete adaptive event-triggered approach (AETA) is introduced to govern signal transmission. With the hope of improving the resource utilization and reducing the update frequency, an event-based fuzzy pinning feedback control strategy is designed to control a small fraction of network nodes. Furthermore, by new Lyapunov–Krasovskii functionals and the finite-time analysis method, sufficient criteria are provided to guarantee the finite-time bounded stability of the closed-loop error system. Under an optimization condition and linear matrix inequality (LMI) constraints, the desired controller parameters with respect to minimum finite time are derived. Finally, several numerical examples are conducted to show the effectiveness of obtained theoretical results. For the same system, the average triggering rate of AETA is significantly lower than existing event-triggered mechanisms and the convergence rate of synchronization errors is also superior to other control strategies.

## 1. Introduction

During the past decades, discrete complex networks (DCNs) have been extensively studied due to the potential advantages of digital simulation and calculation, such as cyber-physical systems [1], multi-agent systems [2,3] and digital communications [4]. Similar to continuous-time complex networks, DCNs are composed of plenty of nodes coupled with edge-to-edge connections where complex dynamic behaviors are included. Hence, studies of the structure, nature and application of DCNs are richly reported in existing literature [5,6,7,8,9]. For instance, Phat et al. designed the switching rule for stability of linear discrete-time systems via LMIs in [5]. The passivity criterion of discrete-time neural networks subject to uncertain parameters was investigated in [6]. Unfortunately, time delays inevitably appear in information transmission between network nodes, which may lead to the oscillatory or instability behavior of coupled networks. Especially in real networked systems, time-varying delays is the problem demanding optimized solutions [10,11,12,13]. In order to eliminate the influence of time-varying coupling delays, a non-fragile protocol was provided for the Markovian jump stochastic system in [11]. The authors discussed switched complex networks with time-varying delays for strictly dissipative conditions in [13]. Therefore, it is a meaningful attempt to analyze dynamical behaviors of DCNs with time-varying delays.

As a significant collective behavior in complex networks, synchronization shows practical significance in a coupled circuit system [14], communication networks [15], genetic networks [16] and industrial internet of things [17] and has become a hot topic of special concern in recent years [18,19,20,21]. For example, the asymptotic synchronization criteria for DCNs were derived under the periodic sampling signals in [19] and the exponential synchronization problem is discussed via topology matrices in [20]. It should be noted that most existing results neglected the time limitation when studying the synchronization behavior of complex networks. Besides, it is extremely difficult to realize complete synchronization (error converges to zero) in practical cases of large-scale complex network structures. Accordingly, the concept of finite-time synchronization is proposed to limit the closed-loop synchronization errors within a certain range in finite time, which has been adopted in related literature [22,23,24,25,26]. In [22,23], the finite-time synchronization problems of switched neural networks affected by delays were solved based on Lyapunov stability theory. The finite-time synchronization conditions are formulated for a class of Markovian jumping complex networks with non-identical nodes and impulsive effects in [24]. Until now, the finite-time boundedness of synchronization error in DCNs is still a challenging issue, which constitutes one of main motivations for our current study.

The Takagi–Sugeno (T-S) fuzzy model is extensively recognized as a powerful tool to deal with a nonlinear system, which can express the nonlinear systems by a set of linear subsystems combined with IF-THEN rules [27,28,29,30]. On one hand, the T-S fuzzy model is used to fuzzify system model for stability analysis. In order to ensure the stability of the closed-loop system, the authors introduced the T-S fuzzy frameworks to the chaotic system in [28]. With regard to delayed Markovian jump complex networks in [30], the T-S fuzzy model was also applied to describing the system nonlinearities. On the other hand, the T-S fuzzy model has been widely applied in controllers. In [31], depending on T-S fuzzy logic, the sampled-data controller was designed to synchronized nodes of reaction–diffusion networks. In order to control complex networks containing communication couplings, Wang et al. proposed the T-S fuzzy feedback controller in [32]. However, a majority of previous results on T-S fuzzy theory concerned the continuous-time system, which prompts us to extend T-S fuzzy model to investigate the finite-time synchronization behaviors of DCNs.

The synchronization control strategy for complex networks has received significant attention [33,34,35]. In view of complex interconnection and huge network scale, it is tough to achieve the desired synchronized state through controlling all network nodes in practice applications. Hence, a pinning control scheme is proposed, which means only part of the nodes need to be directly controlled. As an economical and efficient method, pinning control has been popular in synchronization control. In [36], the pinning synchronization problem of DCNs with time delays was addressed. In the face of partial and discrete-time couplings in networks, the authors designed the pinning sample-data controller in [37]. In addition, the utilization of controller resource is always a focus of concern [38,39]. Recently, along with the advance of digital communication and network techniques, the event-triggered mechanism has been presented to govern the transmission of control signals in practical applications of networked systems, such as sensor networks [40], chaotic circuit networks [41] and multiagent networks [42]. By the event-triggered mechanism, control signals would be updated only if the prespecified triggering condition is satisfied, which means needless resource consumption can be restrained. For example, an event-triggered approach was employed in [43] to design an adaptive sliding mode controller for the stability of a quantized fault system. Furthermore, many efforts are made to improve existing triggering algorithms for less resource consumption. In [44,45], an internal adaptive threshold, also named a dynamic variable, was introduced to form the adaptive event-triggered approach (AETA) to decrease triggering frequency without information packet loss. The related result was also extended to design the state estimator of neural networks in [46]. Based on AETA, energy utilization is further improved in the control process of communication networks and the network congestion is greatly avoided, especially in power systems, wireless networkes and so on. Nevertheless, it is worth noting that finite-time pinning synchronization control for T-S fuzzy DCNs with time-varying delays and couplings under AETA is still a research gap, which motivates us to conduct the study.

Motivated by above discussions, this paper focuses on the finite-time synchronization problem of delayed and coupled TSFDCNs via adaptive event-triggered pinning control strategy. The main contributions of this paper are summarized as follows:

(1) A more general model of DCNs subject to time-varying delays and node couplings is proposed, which extends the existing continuous-time system model and improves the description of discretized dynamic behaviors. By fuzzy membership functions connected by IF-THEN rules, the T-S fuzzy model of DCNs is novelly constructed to analyze the discrete synchronization behaviors;

(2) Based on the adaptive threshold and system errors, a discrete AETA is applied in controller design. By introducing the adaptive triggering condition, the update frequency of control signal is effectively restricted, such that communication resource is saved. Due to the non-negativity of the threshold variable, AETA can decrease the generated event triggering instants compared with static or period triggered mechanisms;

(3) To design effective fuzzy pinning controller, sufficient finite-time synchronization criteria are obtained in terms of LMI constraints and the minimum finite time related optimization condition. According to finite-time control theory and discrete Jensen inequality, less conservative Lyapunov–Krasovskii functionals are established to guarantee the finite-time convergence of synchronization errors;

(4) The effectiveness and generality of the proposed theoretical method are displayed fully. In three various network systems, especially a practical chaotic network, finite-time synchronization can be achieved with fast convergence speed compared with existing methods. Furthermore, it has been shown that the triggering performance of AETA is superior by several comparative experiments.

The rest of this paper is organized as follows: Section 2 provides the formulation of the problem and some requisite preliminaries. Section 3 expounds the main results with proofs of two theorems. Numerical examples are illustrated in Section 4. Finally, Section 5 exhibits the conclusion and outlook.

## 2. Problem Formulation and Preliminaries

In this paper, we consider a class of DCNs with time-varying delays and *N* coupled nodes with the following model:

(1) $$\begin{array}{ll} x_{i}\left(k+1\right)= & Ax_{i}\left(k\right)+B_{1}f\left(x_{i}\left(k\right)\right)+B_{2}h\left(x_{i}\left(k-\tau \left(k\right)\right)\right)\\ & +\;c\sum_{j=1}^{N}g_{ij}\Gamma x_{j}\left(k-\tau \left(k\right)\right)+w_{i}\left(k\right), \end{array}$$

where $x_{i}\left(k\right)=\left[x_{i1}\left(k\right),\;x_{i2}\left(k\right),\;\ldots ,\;x_{in}\left(k\right)\right]\in R^{n}$ denotes the state vector of the *i*th node, $A=diag\{a_{1},\;a_{2},\;\ldots ,\;a_{n}\}$ is real constant matrices, $B_{1}$ and $B_{2}$ are known matrices with appropriate dimensions, *c* represents the coupling strength between nodes. $G={\left(g_{ij}\right)}_{N\times N}$ is the coupled configuration matrix of the network, where $g_{ij}>0$ if there is a connection from *j* to *i* (i≠j), otherwise $g_{ij}=0$. The diagonal elements of matrix *G* are defined as $g_{ii}=-\sum_{j=1,j\neq i}^{N}g_{ij}$, which means $\sum_{j=1}^{N}g_{ij}=0$. $\Gamma \in R^{n}$ is an inner coupling matrix with Γ>0 for i=1, 2, …, N. The exogenous disturbance input w(k) satisfies:

(2) $$\sum_{k=0}^{N}w_{i}^{T}\left(k\right)w_{i}\left(k\right)<\tilde{w}.$$

$f\left(\cdot \right)\in R^{n\times 1}$ and $h\left(\cdot \right)\in R^{n\times 1}$ are nonlinear activation functions of nodes, τ(k) is the time-varying delay with $0<\tau_{m}\leq \tau \left(k\right)\leq \tau_{M}$ for $\tau_{m},\tau_{M}\in N^{+}$. The initial state of system (1) is $x_{i}\left(k\right)=\mu_{i}\left(k\right)$ for $k\in \left\{-\tau_{M},\;-\tau_{M}+1,\;\ldots ,\;0\right\}$.

Suppose $s\left(k\right)\in R^{n}$ is the state of the unforced target node:

(3) $$s\left(k+1\right)=As\left(k\right)+B_{1}f\left(s\left(k\right)\right)+B_{2}h\left(s\left(k-\tau \left(k\right)\right)\right),$$

where $s\left(k\right)={\left(s_{1}\left(k\right),\;s_{2}\left(k\right),\;\ldots ,\;s_{3}\left(k\right)\right)}^{T}\in R^{n}$ represents the state vector of the target node to be synchronized by DCNs (1). f(s(k)) and h(s(k−τ(k))) follow the activation functions given in state Equation (1). s(k)=v(k) denotes the initial value for $k\in {\left[-\tau_{M},0\right]}_{Z}$.

By $e_{i}\left(k\right)$ = $x_{i}\left(k\right)-s\left(k\right)$, the error system is derived as:

(4) $$\begin{array}{c} e_{i}\left(k+1\right)=Ae_{i}\left(k\right)+B_{1}\tilde{f}\left(e_{i}\left(k\right)\right)\;+B_{2}\tilde{h}\left(e_{i}\left(\Delta_{k}\right)\right)+c\sum_{j=1}^{N}g_{ij}\Gamma e_{j}\left(\Delta_{k}\right)+w_{i}\left(k\right), \end{array}$$

where $e_{i}\left(k\right)$ is the synchronization error dynamics between states of network node and target node. $\Delta_{k}=k-\tau \left(k\right)$, $\tilde{f}\left(e_{i}\left(k\right)\right)=f\left(x_{i}\left(k\right)\right)-f\left(s\left(k\right)\right)$, $\tilde{h}\left(e_{i}\left(\Delta_{k}\right)\right)=h\left(x_{i}\left(\Delta_{k}\right)\right)-h\left(s\left(\Delta_{k}\right)\right)$. Due to the existing of node couplings in DCNs, $e_{i}\left(k\right)$ in the error system (4) possesses the same coupling relation for i=1, 2, …, N.

**Remark** **1.** *The states of the presented DCNs and target node contain state vectors, activation functions with and without time delays, which can flexibly describe dynamics of practical systems via changing weight matrices. By assigning the initial values, the dynamic behaviors of s(k) and $x_{i}\left(k\right)$ are determined, such that synchronization errors are measured.*

With the T-S fuzzy model composed of a set of IF-THEN rules, we consider the following fuzzy rule for TSFDCNs:

Fuzzy Rule *l* [22]:

IF $\theta_{1}\left(k\right)$ is $\delta_{1}^{l}$ and … and is $\delta_{p}^{2}$, THEN

(5) $$e_{i}\left(k+1\right)=A_{l}e_{i}\left(k\right)+B_{l1}\tilde{f}\left(e_{i}\left(k\right)\right)\;+B_{l2}\tilde{h}\left(e_{i}\left(\Delta_{k}\right)\right)+c\sum_{j=1}^{N}g_{lij}\Gamma e_{j}\left(\Delta_{k}\right)+w_{i}\left(k\right),$$

where $\theta_{1}\left(k\right),\;\ldots ,\;\theta_{p}\left(k\right)$ are premise variables, $\delta_{1}^{l},\;\ldots ,\;\delta_{p}^{l}$ are fuzzy sets, l∈L={1, 2, …, r}, *r* is the number of fuzzy rules. In order to achieve synchronization, the control strategy is introduced to error system (5). By the weighted average fuzzy inference method, the controlled error system is inferred as:

(6) $$\begin{array}{ll} e_{i}\left(k+1\right) & =\sum_{l=1}^{r}\eta_{l}\left(\theta \left(k\right)\right)\left[A_{l}e_{i}\left(k\right)+B_{l1}\tilde{f}\left(e_{i}\left(k\right)\right)\right.+B_{l2}\tilde{h}\left(e_{i}\left(\Delta_{k}\right)\right)\\ & +c\sum_{j=1}^{N}g_{lij}\Gamma e_{j}\left(\Delta_{k}\right)+\left.w_{i}\left(k\right)\right]+u_{i}\left(k\right), \end{array}$$

where $u_{i}\left(k\right)=\left[u_{i1}\left(k\right),\;u_{i2}\left(k\right),\;\ldots ,\;u_{in}\left(k\right)\right]$ is the control input vector. By means of the technique used in [22,27,29], the normalized membership function $\eta_{l}\left(\theta \left(k\right)\right)$ should satisfy:

$$\eta_{l}\left(\theta \left(k\right)\right)=\frac{\rho_{l}\left(\theta \left(k\right)\right)}{\sum_{l=1}^{r}\rho_{l}\left(\theta \left(k\right)\right)},\;\rho_{l}\left(\theta \left(k\right)\right)=\prod_{j=1}^{p}\delta_{j}^{l}(\theta_{j}\left(k\right)),$$

where $\delta_{j}^{l}\left(\theta_{j}\left(k\right)\right)$ stands for the grade membership of $\theta_{j}\left(k\right)$ in $\delta_{j}^{l}$. Assume that $\rho_{l}\left(\theta \left(k\right)\right)⩾0$, $\sum_{l=1}^{r}\rho_{l}\left(\theta \left(k\right)\right)>0$ for any k⩾0 then we obtain $\eta_{l}\left(\theta \left(k\right)\right)⩾0$ and $\sum_{l=1}^{r}\eta_{l}\left(\theta \left(k\right)\right)=1$.

To improve controller utilization, the following event-triggered condition including adaptive threshold is introduced:

(7) $$\begin{array}{c} k_{s+1}^{i}=\min\left\{k\in N\left|k>k_{s}^{i},\right.\sigma_{i}d_{i}\left(k\right)+\pi_{i}e_{i}^{T}\left(k\right)\Omega_{i}e_{i}\left(k\right)-\varepsilon_{i}^{T}\left(k\right)\Omega_{i}\varepsilon_{i}\left(k\right)<\left.0\right\},\right. \end{array}$$

where $k_{s}^{i}$ is the sth triggered instant of ith node, $k_{0}^{i}=0$, $k_{s+1}^{i}$ is the next triggered instant $\left(k_{s+1}^{i}>k_{s}^{i}\right)$, $\varepsilon_{i}\left(k\right)=e_{i}\left(k_{s}^{i}\right)-e_{i}\left(k\right)$ is the state error between control input updates, $e_{i}\left(k_{s}^{i}\right)$ is the triggered state of error system $e_{i}\left(k_{0}^{i}\right)=e_{i}\left(0\right)$. $\pi_{i}$ and $\sigma_{i}$ are positive constant scalars, $\Omega_{i}$ is a known weighting matrix. The interval adaptive threshold $d_{i}\left(k\right)$ satisfies:

(8) $$\begin{array}{c} d_{i}\left(k+1\right)=\frac{d_{i}\left(k\right)}{{ƛ}_{i}}+\pi_{i}e_{i}^{T}\left(k\right)\Omega_{i}e_{i}\left(k\right)-\varepsilon_{i}^{T}\left(k\right)\Omega_{i}\varepsilon_{i}\left(k\right), \end{array}$$

where ƛ is a given constant, $d_{i}\left(0\right)=d_{i0}⩾0$ is the initial value of $d_{i}\left(k\right)$.

**Remark** **2.** *Based on the dynamic event-triggered mechanism in [40,44], we further propose the adaptive event-triggered condition (7) for the synchronization control of DCNs. Compared with conventional periodic event-triggered and static event-triggered mechanisms, AETA improves the constraint of triggering instants of controller. The event-triggered condition (7) varies in an iterative form by the change of internal adaptive threshold $d_{i}\left(k\right)$. It is obvious that the triggering performance is affected by parameters $\pi_{i}$ and $\sigma_{i}$. The triggering frequency grows as $\sigma_{i}$ becomes closer to zero, while the rise of $\pi_{i}$ leads to the decline of update frequency. Involved in AETA, $\pi_{i}$ and $\sigma_{i}$ can be adjusted flexibly in practical systems and the burden of controller communication will efficiently decrease.*

**Remark** **3.** *The adaptive event-triggered condition is constructed according to synchronization error $e_{i}\left(k\right)$ and absolute error $\varepsilon_{i}\left(k\right)$. In order to simplify the calculation and achieve the quantity analysis of $e_{i}\left(k_{s}^{i}\right)$ within triggering time interval $\left[k_{s}^{i},k_{s+1}^{i}\right)$, $\varepsilon_{i}\left(k\right)$ is measured by $e_{i}\left(k_{s}^{i}\right)-e_{i}\left(k\right)$ to evaluate the absolute error between control updates.*

The control input of the *i*th node shares the same fuzzy rule with the error system (6). Thus, the fuzzy-model-based pinning feedback controller is considered by the following rule:

Fuzzy Rule *l*:

IF $\theta_{1}\left(k\right)$ is $\delta_{1}^{l}$ and … and $\theta_{p}\left(k\right)\;\;$ is $\delta_{p}^{l}$, THEN

(9) $$u_{i}\left(k\right)=-\vartheta_{i}\Pi_{li}e_{i}\left(k_{s}^{i}\right)\;\;\;\;\;\;,\;\;\;\;\;k_{s}^{i}\leq k<k_{s+1}^{i},$$

where $\Pi_{i}$ is the feedback control gain, $\vartheta_{i}$ is the controller parameter. $\vartheta_{i}⩾1$ if the node is pinned, otherwise $\vartheta_{i}=0$. Note that $e_{i}\left(k_{s}^{i}\right)=\varepsilon_{i}\left(k\right)+e_{i}\left(k\right)$, the defuzzified controller $u_{i}\left(k\right)$ can be further described as:

(10) $$u_{i}\left(k\right)=-\sum_{l=1}^{r}\eta_{l}\left(\theta \left(k\right)\right)\left[\vartheta_{i}\Pi_{li}(\varepsilon_{i}\left(k\right)+e_{i}\left(k\right)\right].$$

**Remark** **4.** *In the existing literatures, the T-S fuzzy model is rarely applied to analysis of the dynamical behaviors of DCNs. With a combination of local linear models connected by IF-THEN rules, we novelly propose the model of TSFDCNs, which is the extension of [22,26] and widely appropriate for DCNs analysis. Moreover, the same fuzzy rule is selected to designed the fuzzy pinning feedback controller for closed-loop error system with the hope of reducing computational complexity.*

Substituting the controller (10) to the error system (6), the closed-loop error system of TSFDCNs is obtained. Based on the Kronecker product theory [37,38], we can derive the error system as follows:

(11) $$\begin{array}{ll} e\left(k+1\right) & =\sum_{l=1}^{r}\eta_{l}\left(\theta \left(k\right)\right)\left[A_{l}e\left(k\right)+B_{l1}F\left(k\right)+B_{l2}H\left(\Delta_{k}\right)\right.\\ & +\left.c\left(G_{l}⊗\Gamma \right)e\left(\Delta_{k}\right)+w\left(k\right)-K_{l}\varepsilon \left(k\right)-K_{l}e\left(k\right)\right], \end{array}$$

where

$A_{l}=I_{N}⊗A_{l}$, $B_{l1}=I_{N}⊗B_{l1}$, $B_{l2}=I_{N}⊗B_{l2}$, 

$e\left(k\right)={\left[e_{1}^{T}\left(k\right),\;e_{2}^{T}\left(k\right),\;\ldots ,\;e_{N}^{T}\left(k\right)\right]}^{T}$, 

$\varepsilon \left(k\right)={\left[\varepsilon_{1}^{T}\left(k\right),\;\varepsilon_{2}^{T}\left(k\right),\;\ldots ,\;\varepsilon_{N}^{T}\left(k\right)\right]}^{T}$,

$F\left(k\right)={\left[{\tilde{f}}^{T}(e_{1}\left(k\right),\;{\tilde{f}}^{T}(e_{2}\left(k\right),\;\ldots ,\;{\tilde{f}}^{T}(e_{N}\left(k\right)\right]}^{T}$, 

$H\left(\Delta_{k}\right)=\;{\left[{\tilde{h}}^{T}\left(e_{1}\left(\Delta_{k}\right)\right),\;\;\ldots ,\;{\tilde{h}}^{T}\left(e_{N}\left(\Delta_{k}\right)\right)\right]}^{T}$, 

$w\left(k\right)={\left[w_{1}^{T}\left(k\right),\;w_{2}^{T}\left(k\right),\;\ldots ,\;w_{N}^{T}\left(k\right)\right]}^{T}$, 

$K_{l}=diag\left\{{\tilde{\vartheta }}_{1}\Pi_{l1},\;{\tilde{\vartheta }}_{2}\Pi_{l2},\;\ldots ,\;{\tilde{\vartheta }}_{N}\Pi_{lN}\right\}$. 

The following definition, assumption and lemmas are introduced to discuss synchronization criteria.

**Definition** **1** ([45])**.** *There exist a positive matrix* Φ*, positive constant scalars $m_{1}$, $m_{2}$ $\left(m_{1}<m_{2}\right)$, the TSFDCNs are identified as achieving the finite-time synchronized state with respect to $\left(m_{1},\;m_{2},\;\Phi ,\;\tilde{w},\;T_{m}\right)$ if the error system (11) satisfies:*

(12) $$\begin{array}{c} \left\{\begin{array}{c} \sum_{k=0}^{N}w^{T}\left(k\right)w\left(k\right)<\tilde{w}\\ \underset{k\in \left\{-\tau_{M},-\tau_{M}+1,\ldots ,0\right\}}{\sup}\left\{{\left(\mu (k)-\nu (k)\right)}^{T}\Phi \left(\mu \left(k\right)-\nu \left(k\right)\right)\right\}\leq m_{1} \end{array}\right.\\ \;\;\;\;\;\;\;\;\;\;\;\;\;\;\;\;\;\;\;\;\;\;\;\;\;\;\;\;\;\;\;\;\;\;\;\;\;\;\;\;\;\;\;\;\;\;\;\;\;\;\;\;\Rightarrow e^{T}\left(k\right)\Phi e\left(k\right)<m_{2},\;\;k\in {\left[1,T_{m}\right]}_{Z}. \end{array}$$

**Assumption** **1** ([18])**.** *For all $\iota_{1},\iota_{2},\iota_{3},\iota_{4}\in R^{n}$, it exists following sector-bounded conditions:*

(13) $${\left[f\left(\iota_{1}\right)-f\left(\iota_{2}\right)-U_{1}\left(\iota_{1}-\iota_{2}\right)\right]}^{T}\left[f\left(\iota_{1}\right)-f\left(\iota_{2}\right)-U_{2}\left(\iota_{1}-\iota_{2}\right)\right]\leq 0,$$

(14) $${\left[h\left(\iota_{3}\right)-h\left(\iota_{4}\right)-U_{3}\left(\iota_{3}-\iota_{4}\right)\right]}^{T}\left[h\left(\iota_{3}\right)-h\left(\iota_{4}\right)-U_{4}\left(\iota_{3}-\iota_{4}\right)\right]\;\leq 0,$$

*where node activation functions f(·), h(·) are continuous and satisfy f(0)=0, h(0)=0. $U_{1}$, $U_{2}$, $U_{3}$ and $U_{4}$ are known real matrices with appropriate dimensions.*

**Remark** **5.** *In Assumption 1, (13) and (14) are both referred to a class of sector-bounded condition which is more general than the common Lipschitz continuous condition and are used to restrain system dynamics for bounded continuity. Matrices $U_{1}$, $U_{2}$, $U_{3}$ and $U_{4}$ are given based on functions f(·), h(·).*

**Assumption** **2.** *In order to fully consider the synchronization error dynamics of TSFDCNs, the initial condition of* e(k) *is supposed to satisfy:*

$${\left[e(k+1)-e(k)\right]}^{T}\left[e(k+1)-e(k)\right]\leq \varpi ,$$

*for* $k\in {\left[-\tau_{M},0\right]}_{Z}$ *, where ϖ is a known positive constant.*

**Lemma** **1** ([46])**.** *For a matrix $R\in S_{n}^{+}$, integer a<b and a function p: $Z\left[a,b\right]\to R^{n}$, the following inequalities hold:*

(15) $$\sum_{i=a}^{b}p^{T}\left(i\right)Rp\left(i\right)⩾\frac{1}{\varsigma }\phi_{1}^{T}\bar{R}\phi_{1}$$

(16) $$\sum_{j=a}^{b}\sum_{i=a}^{j}p^{T}\left(i\right)Rp\left(i\right)⩾\frac{2}{\varsigma (\varsigma +1)}\phi_{2}^{T}\tilde{R}\phi_{2},$$

*where* ς=b−a+1*,* $\phi_{1}={\left[\upsilon_{1}^{T},\ell_{1}^{T},\ell_{2}^{T}\right]}^{T}$*,* $\phi_{2}={\left[\upsilon_{2}^{T},\ell_{3}^{T}\right]}^{T}$*,* $\bar{R}=diag\left\{R,3R,5R\right\}$*,* $\tilde{R}=diag\left\{R,8R\right\}$*,* $\ell_{1}=\upsilon_{1}-\frac{2}{\varsigma +1}\upsilon_{2}$*,* $\ell_{2}=\upsilon_{1}-\frac{6}{\varsigma +1}\upsilon_{2}+\frac{12}{\left(\varsigma +1)(\varsigma +2\right)}\upsilon_{3}$*,* $\ell_{3}=\upsilon_{2}-\frac{3}{\varsigma +2}\upsilon_{3}$*,* $\upsilon_{1}\;=\;\sum_{i=a}^{b}p(i)$*,* $\upsilon_{2}=\sum_{j=a}^{b}\sum_{i=a}^{j}p(i)$*,* $\upsilon_{3}=\sum_{℘=a}^{b}\sum_{j=a}^{℘}\sum_{i=a}^{j}p(i)$*.*

**Lemma** **2** ([47])**.** *For given integers n, m, a scalar ℏ∈(0,1), a matrix $J^{n\times n}>0$ and two matrices ${ℵ}_{1},{ℵ}_{2}\in R^{n\times m}$. Define the function χ(ℏ,J) as:*

(17) $$\chi \left(\hbar ,J\right)=\frac{1}{\hbar }\varpi^{T}{ℵ}_{1}^{T}J{ℵ}_{1}\varpi +\frac{1}{1-\hbar }\varpi^{T}{ℵ}_{2}^{T}J{ℵ}_{2}\varpi ,$$

*with all vector* $\varpi \in R^{m}$*. If a matrix* $A\in R^{n\times n}$* such that* $\left[\begin{array}{cc} J & A\\ {}* & J \end{array}\right]>0$* exists, the following inequality holds:*

(18) $$\min_{\hbar \in (0,1)}\chi \left(\hbar ,J\right)⩾{\left[\begin{array}{c} {ℵ}_{1}\varpi \\ {ℵ}_{2}\varpi \end{array}\right]}^{T}\left[\begin{array}{cc} J & A\\ {}* & J \end{array}\right]\left[\begin{array}{c} {ℵ}_{1}\varpi \\ {ℵ}_{2}\varpi \end{array}\right].$$

**Lemma** **3** ([36])**.** *If $x\in R^{n}$, $M\in R^{n\times n}$ is a positive definite matrix, $N\in R^{n\times n}$ is a symmetric matrix, the following inequality is true:*

(19) $$\lambda_{\min}\left(M^{-1}N\right)x^{T}Mx\leq x^{T}Nx\leq \lambda_{\max}\left(M^{-1}N\right)x^{T}Mx.$$

**Lemma** **4.** *For the AETA proposed by (7) and (8), with the initial value $d_{i0}⩾0$, the adaptive threshold parameter $d_{i}\left(k\right)$ will be non-negative for ∀k⩾0 if condition 0<σƛ≤1 is satisfied where $\sigma_{i}\in \left(0,1\right)$ and ${ƛ}_{i}>1$.*

**Proof** **of Lemma 4.** Based on the definition of event-triggered condition (7), it is easy to get $\sigma_{i}d_{i}\left(k\right)+\pi_{i}e_{i}^{T}\left(k\right)\Omega_{i}e_{i}\left(k\right)-\varepsilon_{i}^{T}\left(k\right)\Omega_{i}\varepsilon_{i}\left(k\right)⩾0$, ∀k⩾0 when system is controlled, which derives that:

$$-\sigma_{i}d_{i}\left(k\right)\leq \pi_{i}e_{i}^{T}\left(k\right)\Omega_{i}e_{i}\left(k\right)-\varepsilon_{i}^{T}\left(k\right)\Omega_{i}\varepsilon_{i}\left(k\right).$$

Then, from (8), we can further obtain:

$$\begin{array}{c} d_{i}\left(k+1\right)=\frac{d_{i}\left(k\right)}{\;{ƛ}_{i}}+\pi_{i}e_{i}^{T}\left(k\right)\Omega_{i}e_{i}\left(k\right)-\varepsilon_{i}^{T}\left(k\right)\Omega_{i}\varepsilon_{i}\left(k\right)\\ \;\;\;\;\;\;\;\;\;\;\;\;\;\;\;\;\;\;\;\;\;\;\;\;\;\;\;\;\;\;\;\;\;⩾\left(1/{ƛ}_{i}-\sigma_{i}\right)d_{i}\left(k\right)\\ \;\;\;\;\;\;\;\;\;\;\;\;\;\;\;\;\;\;\;\;\;\;\;\;\;\;\;\;\;\;\;\;\;⩾{\left(1/{ƛ}_{i}-\sigma_{i}\right)}^{2}d_{i}\left(k-1\right)\\ \;\;\;\;\;\;\;\;\;\;\;\;\;\;\;\;\;\;\;\;\;\;\;\;\;\;\;\;\;\;\;\;\;\;\vdots \\ \;\;\;\;\;\;\;\;\;\;\;\;\;\;\;\;\;\;\;\;\;\;\;\;\;\;\;\;\;\;\;\;\;⩾{\left(1/{ƛ}_{i}-\sigma_{i}\right)}^{k+1}d_{i0}. \end{array}$$

If conditions of $0<\sigma_{i}{ƛ}_{i}\leq 1$ and $d_{i0}>0$ are satisfied, $d_{i}\left(k\right)⩾0$ will hold for any k⩾0. □

**Remark** **6.** *For event-triggered mechanism, signal transmits only when established condition is satisfied. By Lemma 4, the non-negativity of $d_{i}\left(k\right)$ is guaranteed for all k⩾0, such that it is unnecessary to ensure the inequation $\pi_{i}e_{i}^{T}\left(k\right)\Omega_{i}e_{i}\left(k\right)-\varepsilon_{i}^{T}\left(k\right)\Omega_{i}\varepsilon_{i}\left(k\right)⩾0$ holding all the time when synchronization is reached, which relaxes the conditions in static or period event-triggered mechanisms. Therefore, the controller triggering frequency is reduced.*

## 3. Main Results

In this section, several sufficient conditions are analyzed for finite-time synchronization of TSFDCNs.

### 3.1. Pinning Finite-Time Synchronization for TSFDCNs with Time-Varying Delays

**Theorem** **1.** Assume that $\sigma_{i}\in \left(0,1\right)$ and ${ƛ}_{i}>1$ satisfy $\sigma_{i}{ƛ}_{i}\leq 1$. For given positive constant scalars $m_{1}<m_{2}$, ϖ>1, y>1, a matrix Φ>0, the TSFDCNs will be finite-time synchronized with respect to $\left(m_{1},m_{2},\Phi ,\tilde{w},T_{m}\right)$ if there exist symmetric matrix $Q=\mathrm{diag}\left\{Q_{1},\;Q_{2},\;\ldots ,\;Q_{N}\right\}$, $K_{l}=\mathrm{diag}\left\{K_{l1},\;K_{l2},\;\ldots ,\;K_{lN}\right\}$, $\Omega =\mathrm{diag}\left\{\Omega_{1},\;\Omega_{2},\;\ldots ,\;\Omega_{N}\right\}\in R^{nN\times nN}$, positive definite matrices $\Upsilon_{1}$, $\Upsilon_{2}$, $\Upsilon_{3}$, $\Upsilon_{4}$, $\Upsilon_{5}\in R^{nN\times nN}$, positive constant scalars $o_{i}\left(i=1,2,3,4\right)$, $\lambda_{i}\left(i=0,1,2,3,4,5\right)$, $\bar{w}$, $\hbar^{*}$, $\hbar_{1}$, $\hbar_{2}$ and a matrix $R\in R^{3nN\times 3nN}$ satisfying:

(20) $$\begin{array}{cc} \; & \left[\begin{array}{cc} {\tilde{\Upsilon }}_{3} & R\\ {}* & {\tilde{\Upsilon }}_{3} \end{array}\right]>0,\\ \; & \lambda_{0}I\leq Q^{*}\leq \lambda_{1}I,\;0\leq \Upsilon_{1}^{*}\leq \lambda_{2}I,\;0\leq \Upsilon_{2}\leq \lambda_{3}I,\\ \; & 0\leq \Upsilon_{3}\leq \lambda_{4}I,\;0\leq \Upsilon_{4}\leq \lambda_{5}I,\;0\leq \Upsilon_{5}\leq \bar{w}I,\\ \; & \left[\begin{array}{cc} \Psi_{1} & \Psi_{2}\\ {}* & -\Theta^{-1} \end{array}\right]<0,\\ \; & L\leq m_{2}\left(1-y^{-1}\right),\\ \; & m_{1}L_{1}+\varpi L_{2}+y\sum_{i=1}^{N}\sigma_{i}d_{i0}+\tilde{w}\bar{w}\leq \lambda_{0}y^{T_{m}}m_{2}, \end{array}$$

*where *

$\Psi_{1}=\left[\begin{array}{ccc} J_{11} & 0 & J_{13}\\ {}* & J_{22} & 0\\ {}* & * & J_{33} \end{array}\right],$

$\Psi_{2}=\left[A_{l}-K_{l}-I_{nN},c\left(G_{l}⊗\Gamma \right),\underset{8}{\underbrace{0,0,\cdots ,0}},B_{l1},B_{l2},I_{nN},0,K_{l}\right],$

$\Theta =Q+\frac{\tau_{m}\left(\tau_{m}+1\right)}{2}\Upsilon_{2}+{\left(\tau_{M}-\tau_{m}\right)}^{2}\Upsilon_{3}+\tau_{m}^{2}\Upsilon_{4},$

$\begin{array}{c} J_{11}\;=\;\Xi_{1}\left[2K_{l}-\left(1+y^{-1}\right)Q+\left(\tau_{M}-\tau_{m}+1\right)\Upsilon_{1}+yℑ\Omega \right]\Xi_{1}^{T}+y^{-\tau_{M}}\Xi_{2}\Upsilon_{1}\Xi_{2}^{T}\\ \;\;\;\;\;\;\;\;\;\;\;\;\;\;\;\;\;\;\;\;\;\;+\mathrm{Sym}\left\{\Xi_{1}QA_{l}\Xi_{1}^{T}+\Xi_{1}QB_{l1}\Xi_{11}^{T}+\Xi_{1}QB_{l2}\Xi_{12}^{T}\right.\left.+c\Xi_{1}Q\left(G_{l}⊗\Gamma \right)\Xi_{2}^{T}+\Xi_{1}Q\Xi_{13}^{T}\right\}\\ \;\;\;\;\;\;\;\;\;\;\;\;\;\;\;\;\;\;\;\;\;\;-\Xi_{13}\Upsilon_{5}\Xi_{13}^{T}-\frac{y^{-1}}{\tau_{M}-\tau_{m}}\Lambda_{2}{\tilde{\Upsilon }}_{2}\Lambda_{2}^{T}+y^{\tau_{m}+1}\Lambda_{2}\left[\begin{array}{cc} {\tilde{\Upsilon }}_{3} & R\\ & {\tilde{\Upsilon }}_{3} \end{array}\right]\Lambda_{2}^{T}-\Lambda_{3}{\tilde{\Upsilon }}_{4}\Lambda_{3}^{T}\\ \;\;\;\;\;\;\;\;\;\;\;\;\;\;\;\;\;\;\;\;\;\;-\hbar_{1}\Lambda_{4}A\Lambda_{4}^{T}-\hbar_{2}\Lambda_{5}M\Lambda_{5}^{T}, \end{array}$

$\begin{array}{c} J_{22}=\mathrm{diag}\left\{\sigma_{1}\left(\frac{y}{\;{ƛ}_{1}}-1+\hbar^{*}\right),\sigma_{2}\left(\frac{y}{\;{ƛ}_{2}}-1+\hbar^{*}\right),\ldots ,\sigma_{N}\left(\frac{y}{\;{ƛ}_{N}}-1+\hbar^{*}\right)\right\} \end{array}$

$J_{13}=-\Xi_{1}K_{l}$, $\begin{array}{c} J_{33}=\mathrm{diag}\left\{-\left(\sigma_{1}y+\hbar^{*}\right)\Omega_{1},-\left(\sigma_{2}y+\hbar^{*}\right)\Omega_{2},\ldots ,-\left(\sigma_{N}y+\hbar^{*}\right)\Omega_{N}\right\} \end{array}$

$ℑ=\mathrm{diag}\left\{\sigma_{1}\pi_{1},\sigma_{2}\pi_{2},\ldots ,\sigma_{2}\pi_{2}\right\},$

$\Lambda_{1}=\left[\Xi_{2}-\Xi_{7},\Xi_{2}-4\Xi_{7}-\Xi_{10},\Xi_{3}-\Xi_{8},\Xi_{3}-4\Xi_{8}+3\Xi_{11}\right],$

$\begin{array}{c} \Lambda_{2}=[\Xi_{4}-\Xi_{2},\Xi_{4}-\Xi_{2}-2\Xi_{7},\Xi_{4}-\Xi_{2}+6\Xi_{7}-6\Xi_{10},\Xi_{2}-\Xi_{3},\\ \;\;\;\;\;\;\;\;\;\;\;\;\;\;\;\;\;\;\;\;\;\;\;\;\;\;\;\;\;\;\;\;\;\;\;\;\;\;\;\;\;\;\;\;\;\;\;\;\;\;\;\;\;\;\;\;\Xi_{2}-\Xi_{3}-2\Xi_{7},\Xi_{2}-\Xi_{3}+6\Xi_{8}-6\Xi_{11}]\;, \end{array}$

$\Lambda_{3}=\left[\Xi_{1}-\Xi_{4},\Xi_{1}+\Xi_{4}-2\Xi_{7},\Xi_{1}-\Xi_{4}+6\Xi_{6}-6\Xi_{9}\right],$

$\Lambda_{4}={\left[\begin{array}{cc} \Xi_{1} & \Xi_{11} \end{array}\right]}^{T},$ $\Lambda_{5}={\left[\begin{array}{cc} \Xi_{1} & \Xi_{12} \end{array}\right]}^{T},$

$Q^{*}=\Phi^{-1/2}Q\Phi^{-1/2}$ *,* $\Upsilon_{1}^{*}=\Phi^{-1/2}\Upsilon_{1}\Phi^{-1/2},$

$\Xi_{i}=\left[\begin{array}{ccc} 0_{nN\times (i-1)nN} & I_{nN} & 0_{nN\times (15-i)nN} \end{array}\right],$

$L_{1}=\lambda_{1}+o_{1}\lambda_{2},$ $L_{2}=o_{2}\lambda_{3}+\left(\tau_{M}-\tau_{m}\right)o_{3}\lambda_{4}+\tau_{m}o_{4}\lambda_{5},$

$o_{1}=\frac{y^{-\tau_{m}}-1}{y^{-1}-1},$

$\begin{array}{c} o_{2}=\frac{y^{-\tau_{M}-2}-y^{-\tau_{m}-2}+y^{-1}\left(\tau_{M}-\tau_{m}\right)\left(\tau_{M}+\tau_{m}+2\right)}{{\left(y^{-1}-1\right)}^{3}}\\ \;\;\;\;\;\;\;\;\;\;\;\;\;\;\;\;\;\;-\frac{\left(\tau_{M}-\tau_{m}\right)\left[y^{-2}\left(\tau_{M}+\tau_{m}+3\right)-\left(\tau_{M}+\tau_{m}+1\right)\right]}{2{\left(y^{-1}-1\right)}^{3}},\; \end{array}$

$o_{3}=\frac{y^{-\tau_{M}-1}-y^{-\tau_{m}-1}-\left(\tau_{M}-\tau_{m}\right)y^{-1}+\tau_{M}-\tau_{m}}{{\left(y^{-1}-1\right)}^{2}}$ *,* $o_{4}=\frac{y^{-\tau_{m}-1}-\left(\tau_{m}+1\right)y^{-1}+\tau_{m}}{{\left(y^{-1}-1\right)}^{2}}.$

*Besides, the desired gains matrix of the controller is designed by:*

(21) $$K_{li}=Q_{i}^{-1}K_{li},\;i=1,\;2,\;\ldots ,\;N.$$

**Proof of Theorem 1.**  The detailed proof is provided in Appendix A. □

**Remark** **7.** *By Theorem 1, we first propose an event-based framework to analyze the finite-time pinning synchronization issue for a class of time-varying delayed TSFDCNs. Based on the finite time control technique, sufficient criteria to guarantee the stability of the closed-loop error system are derived via building Lyapunov–Krasovskii functionals, which covers more error and delay information to reduce the conservativeness. Meanwhile, Theorem 1 developed an optimization algorithm with respect to minimum finite time $T_{m}$ of achieving synchronization based on $m_{2}$ and adaptive event-triggered threshold $\sigma_{i}d_{i}\left(k\right)$. Solving the LMIs in (20), gains of the desired T-S fuzzy pinning controller can be derived based on $Q_{i}$ and $K_{li}$, which extends efficient methods in the literature [18,22,26]. Obviously, the computational complexity of the algorithm depends on the number of coupled nodes.*

**Remark** **8.** *To guarantee the lower conservativeness of proposed theoretical results, a Lyapunov–Krasovskii functional candidate containing more system information is established. $V_{2}\left(k\right)$ is introduced to capture the variation of adaptive threshold $\sigma_{i}d_{i}\left(k\right)$, which promotes the effectiveness of the controller. Compared with stability analysis in References [34,44], new terms $V_{4}\left(k\right)$ and $V_{5}\left(k\right)$ are designed to ensure the stability of absolute error β(k), such that the synchronization performance is further improved. In addition, a class of discrete Jensen inequality proposed by Lemma 1 can approximate the range of Lyapunov terms more accurately.*

### 3.2. Pinning Finite-Time Synchronization for DCNs

**Definition** **2.** *There exist a positive matrix* Φ *and positive constants $m_{1}$, $m_{2}$ $\left(m_{1}<m_{2}\right)$, the DCNs are identified as achieving the finite-time synchronized state with respect to $\left(m_{1},m_{2},\Phi ,T_{m}\right)$ if the error system (46) satisfies:*

(22) $$\begin{array}{c} \underset{k\in \left\{-\tau ,-\tau +1,\ldots 0\right\}}{\sup}\left\{{\left(\mu (k)-\nu (k)\right)}^{T}\Phi \left(\mu \left(k\right)-\nu \left(k\right)\right)\right\}\leq m_{1}\\ \;\;\;\;\;\;\;\;\;\;\;\;\;\;\;\;\;\;\;\;\;\;\;\;\;\;\;\;\;\;\;\;\;\;\;\;\;\;\;\;\;\;\;\;\;\;\;\;\;\;\;\;\;\;\;\;\;\Rightarrow e^{T}\left(k\right)\Phi e\left(k\right)<m_{2}\;\;\;,k\in \left[1,T_{m}\right]. \end{array}$$

Consider a case where the T-S fuzzy model is not involved and the complex networks are influenced by constant time delay τ—the corresponding error system can be described as:

(23) $$e\left(k+1\right)\;=Ae\left(k\right)+B_{1}F\left(k\right)+B_{2}H\left(\Delta_{\tau }\right)+c\left(G⊗\Gamma \right)e\left(\Delta_{\tau }\right)-K\varepsilon \left(k\right)-Ke\left(k\right),$$

where $\Delta_{\tau }=k-\tau$. By the model (50), we are going to derive a new result on finite-time synchronization control for DCNs.

**Theorem** **2.** Assume that $\sigma_{i}\left(0<\sigma_{i}<1\right)$ and ${ƛ}_{i}\left({ƛ}_{i}>1\right)$ satisfy $\sigma_{i}{ƛ}_{i}\leq 1$. For given positive scalars $m_{1}<m_{2}$, ϖ>1, y>1, a matrix Φ>0, the DCNs will be finite-time synchronized with respect to $\left(m_{1},\;m_{2},\;\Phi ,\;T_{m}\right)$ if there exists a symmetric matrix $Q=diag\left\{Q_{1},\;Q_{2},\;\ldots ,\;Q_{N}\right\}$, $K=diag\left\{K_{1},\;K_{2},\;\ldots ,\;K_{N}\right\}$, $\Omega =\mathrm{diag}\left\{\Omega_{1},\;\Omega_{2},\;\ldots ,\;\Omega_{N}\right\}\in R^{nN\times nN}$, positive definite matrices $\Upsilon_{1}$, $\Upsilon_{2}$, $\Upsilon_{3}$, positive constants ${\tilde{o}}_{i}\left(i=1,2,3\right)$, ${\tilde{\lambda }}_{i}\left(i=0,1,2,3,4\right)$, $\hbar^{*}$, $\hbar_{1}$, $\hbar_{2}$ and a matrix $R\in R^{3nN\times 3nN}$ satisfying:

(24) $$\begin{array}{cc} \; & \lambda_{0}I\leq Q^{*}\leq \lambda_{1}I,\;0\leq \Upsilon_{1}^{*}\leq \lambda_{2}I,\\ \; & 0\leq \Upsilon_{2}\leq \lambda_{3}I,\;0\leq \Upsilon_{3}\leq \lambda_{4}I,\\ \; & \left[\begin{array}{cc} {\tilde{\Psi }}_{1} & {\tilde{\Psi }}_{2}\\ {}* & -{\tilde{\Theta }}^{-1} \end{array}\right]<0,\\ & L\leq m_{2}\left(1-y^{-1}\right),\\ \; & m_{1}{\tilde{L}}_{1}+\varpi {\tilde{L}}_{2}+y\sum_{i=1}^{N}\sigma_{i}d_{i0}\leq y^{T_{m}}\lambda_{0}m_{2}, \end{array}$$

*where*

${\tilde{\Psi }}_{1}=\left[\begin{array}{cccccccc} H_{11} & H_{12} & H_{13} & H_{14} & H_{15} & H_{16} & 0 & H_{18}\\ {}* & H_{22} & H_{23} & H_{24} & 0 & 0 & 0 & 0\\ {}* & * & H_{33} & H_{34} & 0 & 0 & 0 & 0\\ {}* & * & * & H_{44} & 0 & 0 & 0 & 0\\ {}* & * & * & * & H_{55} & 0 & 0 & 0\\ {}* & * & * & * & * & H_{66} & 0 & 0\\ {}* & * & * & * & * & * & H_{77} & 0\\ {}* & * & * & * & * & * & * & H_{88} \end{array}\right]$,

${\tilde{\Psi }}_{2}=\left[A-K-I_{nN},c\left(G⊗\Gamma \right),0,0,B_{1},B_{2},0,K\right]$,

$\tilde{\Theta }=Q+\tau^{2}\Upsilon_{2}+\frac{\tau (\tau +1)}{2}\Upsilon_{3}$,

$\begin{array}{c} H_{11}=-\left(1+y^{-1}\right)+\Upsilon_{1}+\Upsilon_{2}+3z_{1}\left(\tau \right)\Upsilon_{2}+5z_{2}\left(\tau \right)\Upsilon_{2}+2QA\\ \;\;\;\;\;\;\;\;\;\;\;\;\;\;\;\;\;\;\;-2K+yℑ\Omega -\hbar_{1}A_{1}-\hbar_{2}M_{1}, \end{array}$

$H_{12}=-\Upsilon_{2}+3z_{1}\left(\tau \right)\Upsilon_{2}-5z_{2}\left(\tau \right)\Upsilon_{2}+cQ\left(G⊗\Gamma \right)$, $H_{13}=-6z_{1}\left(\tau \right)\Upsilon_{2}+30z_{2}\left(\tau \right)\Upsilon_{2}$,

$H_{14}=-30z_{2}\left(\tau \right)\Upsilon_{2}$*,*$H_{15}=QB_{1}-\hbar_{1}A_{2}$*,*$H_{16}=QB_{2}-\hbar_{2}M_{2}$,

$H_{18}=K$*,*$H_{22}=-y^{-\tau }+\Upsilon_{2}+3z_{1}\left(\tau \right)\Upsilon_{2}-5z_{2}\left(\tau \right)\Upsilon_{2}+\Upsilon_{3}+2z_{3}\left(\tau \right)\Upsilon_{3}$,

$H_{23}=-6z_{1}\left(\tau \right)\Upsilon_{2}-30z_{2}\left(\tau \right)\Upsilon_{2}-4\Upsilon_{3}-8z_{3}\left(\tau \right)\Upsilon_{3}$*,*$H_{24}=30z_{2}\left(\tau \right)\Upsilon_{2}+6z_{3}\left(\tau \right)\Upsilon_{3}$,

$H_{33}=12z_{1}\left(\tau \right)\Upsilon_{2}+180z_{2}\left(\tau \right)\Upsilon_{2}+16\Upsilon_{3}+32z_{3}\left(\tau \right)\Upsilon_{3}$*,*$H_{34}=-180z_{2}\left(\tau \right)\Upsilon_{2}-24z_{3}\left(\tau \right)\Upsilon_{3}$,

$H_{44}=180z_{2}\left(\tau \right)\Upsilon_{2}+18z_{3}\left(\tau \right)\Upsilon_{3}$*,*$H_{55}=-\hbar_{1}I_{N}$*,*$H_{66}=-\hbar_{2}I_{N}$,

$H_{77}=J_{22}$*and*$H_{88}=J_{33}$*are defined in (21),*,

$Q^{*}=\Phi^{-1/2}Q\Phi^{-1/2}$*,*$\Upsilon_{1}^{*}=\Phi^{-1/2}\Upsilon_{1}\Phi^{-1/2}$,

${\tilde{L}}_{1}=\lambda_{1}+{\tilde{o}}_{1}\lambda_{2}$*,*${\tilde{L}}_{2}={\tilde{o}}_{2}\lambda_{3}+{\tilde{o}}_{3}\lambda_{4}$,

${\tilde{o}}_{1}=\frac{y^{-\tau }-1}{y^{-1}-1}$*,*${\tilde{o}}_{2}=\frac{y^{-\tau -1}-\left(\tau +1\right)y^{-1}+\tau }{{\left(y^{-1}-1\right)}^{2}}$,

${\tilde{o}}_{3}=\frac{y^{-\tau -2}}{{\left(y^{-1}-1\right)}^{3}}-\frac{y^{-2}\left(\tau +1\right)\left(\tau +2\right)}{2{\left(y^{-1}-1\right)}^{3}}+\frac{y^{-1}\left(2\tau +\tau^{2}\right)}{{\left(y^{-1}-1\right)}^{3}}-\frac{\tau +\tau^{\tau }}{2{\left(y^{-1}-1\right)}^{3}},$

*and the controller gains matrix is given by:*

(25) $$K_{i}=Q_{i}^{-1}K_{i},\;i=1,\;2,\;\ldots ,\;N.$$

**Proof of Theorem 2.**  The detailed proof is provided in Appendix B. □

**Remark** **9.** *Theorem 2 is the development of Theorem 1, which can also be regarded as the discrete counterpart of Corollary 1 in [22], as well as the extension of results in [9]. From Definitions 1 and 2, we get the finite-time analysis method of synchronization dynamics, which differs from traditional asymptotic synchronization. Rather than reaching mean-square stable, e(k) converges to the certain region $\left\{e\left|e^{T}\left(k\right)\Phi e\left(k\right)<m_{2}\right.\right\}$ only if sufficiently small $T_{m}$ and sufficiently large $m_{2}$ exist, which brings a certain degree of freedom.*

**Remark** **10.** *In the existing literature, fruitful achievements on the synchronization and stability control of complex networks are reported [11,16,22,28,34,37]. T-S fuzzy sampled-data control was applied to guarantee the finite-time synchronization of switched complex networks in [22] and the stability of chaotic systems in [28]. Exponential synchronization of delayed complex networks was investigated in [34]. Compared with most results, this paper presents the following novel technologies: (1) the T-S fuzzy model is involved to establish DCNs for discrete dynamical analysis; (2) the finite-time pinning synchronization control is the first attempt for TSFDCNs under AETA; (3) new criteria including optimization conditions are proposed to guarantee the finite-time boundedness of the error system.*

## 4. Numerical Experiments

In this section, numerical examples are provided to illustrate the effectiveness of the proposed synchronization strategy.

**Example** **1.** *Based on the IF-THEN rules, the TSFDCNs consisting of five nodes (N = 5) are considered as follows:*

***Rule 1.***  *IF* $\theta_{1}\left(k\right)\;\;$ is $\delta_{p}^{1}$*, THEN*

$\begin{array}{c} x_{i}\left(k+1\right)=A_{1}x_{i}\left(k\right)+B_{11}f\left(x_{i}\left(k\right)\right)+B_{12}h\left(x_{i}\left(\Delta_{k}\right)\right)\\ \;\;\;\;\;\;\;\;\;\;\;\;\;\;\;\;\;\;\;\;\;\;\;\;\;\;\;\;\;\;\;\;\;\;\;\;\;\;\;\;\;\;\;\;+cG_{1}⊗\Gamma_{1}x_{j}\left(\Delta_{k}\right)+w_{i}\left(k\right)\;, \end{array}$

***Rule 2.*** *IF*$\theta_{2}\left(k\right)\;\;$*is*$\delta_{p}^{2}$*, THEN*

$\begin{array}{c} x_{i}\left(k+1\right)=A_{2}x_{i}\left(k\right)+B_{21}f\left(x_{i}\left(k\right)\right)+B_{22}h\left(x_{i}\left(\Delta_{k}\right)\right)\\ \;\;\;\;\;\;\;\;\;\;\;\;\;\;\;\;\;\;\;\;\;\;\;\;\;\;\;\;\;\;\;\;\;\;\;\;\;\;\;\;\;\;\;\;+cG_{2}⊗\Gamma_{2}x_{j}\left(\Delta_{k}\right)+w_{i}\left(k\right)\;.\;\;\;\;\;\; \end{array}$

**Figure 1:**
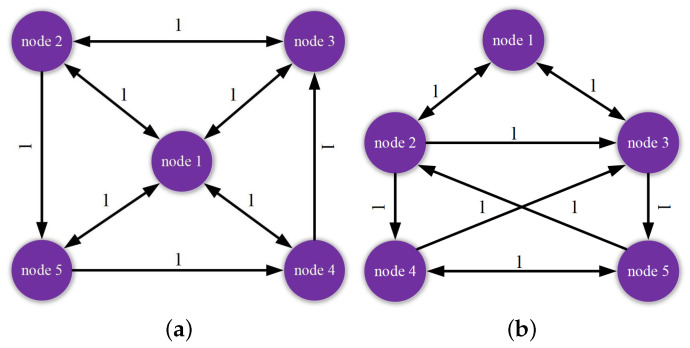
Communication coupling structure for two fuzzy rules. (**a**) Rule 1. (**b**) Rule 2.

The membership functions of Rule 1 and Rule 2 are defined as $\eta_{1}\left(\theta \left(k\right)\right)=1-\sin^{2}\left(\frac{k}{2}\right)$ and $\eta_{2}\left(\theta \left(k\right)\right)=1-\eta_{1}\left(\theta \left(k\right)\right)$ respectively. From the directed topological structures shown in Figure 1, the coupled configuration matrices $G_{1}$ and $G_{2}$ of two fuzzy rules are chosen as:

$$G_{1}=\left[\begin{array}{ccccc} -3 & 1 & 1 & 1 & 1\\ 1 & -2 & 1 & 0 & 1\\ 1 & 1 & -2 & 0 & 0\\ 1 & 0 & 1 & -2 & 0\\ 1 & 0 & 0 & 1 & -2 \end{array}\right],\;G_{2}=\left[\begin{array}{ccccc} -4 & 1 & 1 & 0 & 0\\ 1 & -2 & 1 & 1 & 0\\ 1 & 1 & -3 & 0 & 1\\ 0 & 0 & 1 & -2 & 1\\ 0 & 1 & 0 & 1 & -2 \end{array}\right]$$.

Some parameters are assumed as:

$A_{1}=A_{2}=0.8I_{2}$, $B_{11}=B_{21}=\left[\begin{array}{cc} -1 & 0.5\\ 0.5 & -1 \end{array}\right]$, $B_{12}=B_{22}=\left[\begin{array}{cc} -0.3 & 0.25\\ 0 & 0.2. \end{array}\right]$. 

The nonlinear activation functions of TSFDCNs are:

$f\left(x_{i}\left(k\right)\right)=\left[\begin{array}{c} 1.85x_{1}\left(k\right)+0.25x_{2}\left(k\right)+\tanh\left(0.05x_{1}\left(k\right)\right)\\ -0.35x_{2}\left(k\right)-\tanh\left(0.05x_{1}\left(k\right)+0.05x_{2}\left(k\right)\right) \end{array}\right]$, 

$h\left(x_{i}\left(\Delta_{k}\right)\right)=\left[\begin{array}{c} -0.3x_{1}\left(\Delta_{k}\right)+0.5x_{2}\left(\Delta_{k}\right)+\tanh\left(0.5x_{1}\left(\Delta_{k}\right)+0.5x_{2}\left(\Delta_{k}\right)\right)\\ -0.4x_{2}\left(\Delta_{k}\right)-\tanh(0.2x_{1}\left(\Delta_{k}\right) \end{array}\right]$.

By Assumption 1, select:

$U_{1}=\left[\begin{array}{cc} 1.85 & 0.25\\ -0.25 & 0.6 \end{array}\right]$, $U_{2}=\left[\begin{array}{cc} 0.4 & 0.8\\ 0 & -0.35 \end{array}\right]$

$U_{3}=\left[\begin{array}{cc} -0.3 & 0.5\\ 0.75 & 0.9 \end{array}\right]$, $U_{4}=\left[\begin{array}{cc} -0.15 & 0.2\\ 0 & -0.4. \end{array}\right]$

The time-varying delay is taken as τ(k)=1+2sin2(kπkπ22), where $\tau_{m}=1$, $\tau_{M}=3$ ([a] denotes the integer part of the number a), the exogenous disturbance is set as wi(k)=0.6e0.1ksin(k)0.6e0.1ksin(k)(1+e0.1k)(1+e0.1k), 0.6e0.01kcos(k)0.6e0.01kcos(k)(1+e0.01k)(1+e0.01k)T. Let parameters c=1.2, matrices $\Gamma_{1}=\Gamma_{2}=diag\left\{-1.25,-0.85\right\}$.

**Figure 2:**
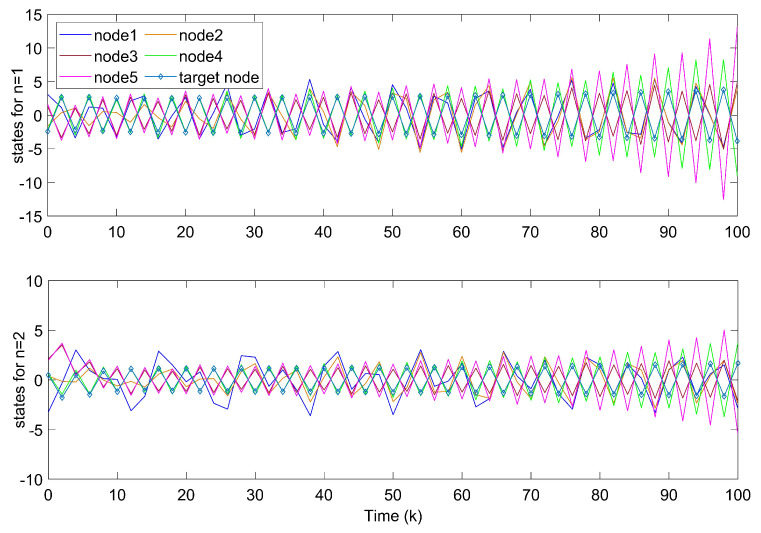
States of nodes $x_{i1},\;x_{i2}$ in TSFDCNs.

**Figure 3:**
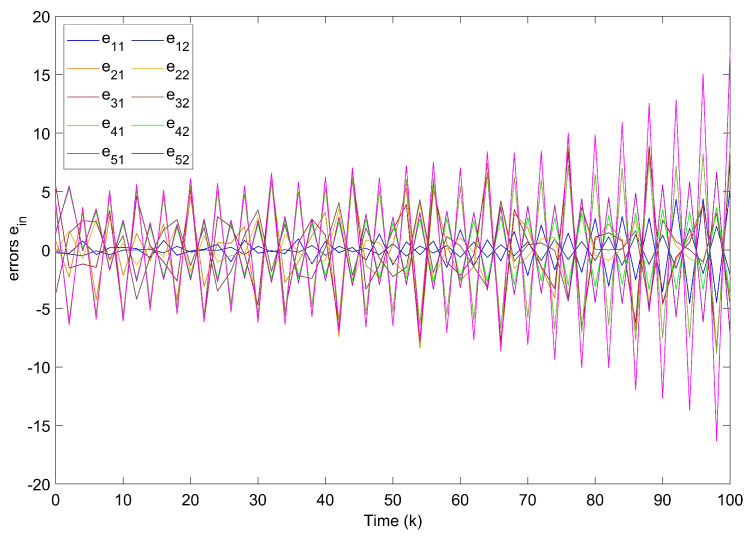
Synchronization errors $e_{in}$ without controllers of TSFDCNs.

Shown in Figure 2, the system fails to track the motion of the target node without controllers. In Figure 3, state errors of nodes in TSFDCNs tend to diverge with time, which implies that the desired synchronization cannot be achieved.

According to Theorem 1, some parameters are chosen as Φ=I, $m_{1}=15$, $m_{2}=200$, $T_{m}=50$, $\tilde{w}=0.36$, $\hbar^{*}=1$, $\hbar_{1}=\hbar_{2}=0.8$. For adaptive event-triggered condition (7), we set Ω=I, π=0.5, σ=0.6, ƛ=1.5 and $d_{i0}=0.1$. Solving the LMIs in Theorem 1, we obtain the following control gains $\Pi_{li}$ under fuzzy rules 1 and 2 when all nodes are controlled:

$\Pi_{11}=\left[\begin{array}{cc} 0.0078 & -1.3824\\ -1.7081 & -0.2099 \end{array}\right]$, $\Pi_{12}=\left[\begin{array}{cc} 0.0263 & -1.8317\\ -1.6910 & -0.2821 \end{array}\right]$, 

$\Pi_{21}=\left[\begin{array}{cc} 0.2476 & -1.6201\\ -1.8991 & -1.1850 \end{array}\right]$, $\Pi_{22}=\left[\begin{array}{cc} 0.1263 & -1.2317\\ -1.2501 & -1.8925 \end{array}\right]$, 

$\Pi_{31}=\left[\begin{array}{cc} 0.0135 & -1.4855\\ -1.7287 & -1.0990 \end{array}\right]$, $\Pi_{32}=\left[\begin{array}{cc} 0.3829 & -1.6715\\ -1.9872 & -1.2430 \end{array}\right]$,

$\Pi_{41}=\left[\begin{array}{cc} 0.1147 & -1.7001\\ -1.9306 & -2.0012 \end{array}\right]$, $\Pi_{42}=\left[\begin{array}{cc} 0.5772 & -1.9144\\ -1.7668 & -2.4312 \end{array}\right]$

$\Pi_{51}=\left[\begin{array}{cc} 0.1783 & -1.5103\\ -2.5691 & -1.1975 \end{array}\right]$, $\Pi_{52}=\left[\begin{array}{cc} 0.2839 & -1.0769\\ -2.0657 & -1.9128 \end{array}\right]$.

**Figure 4:**
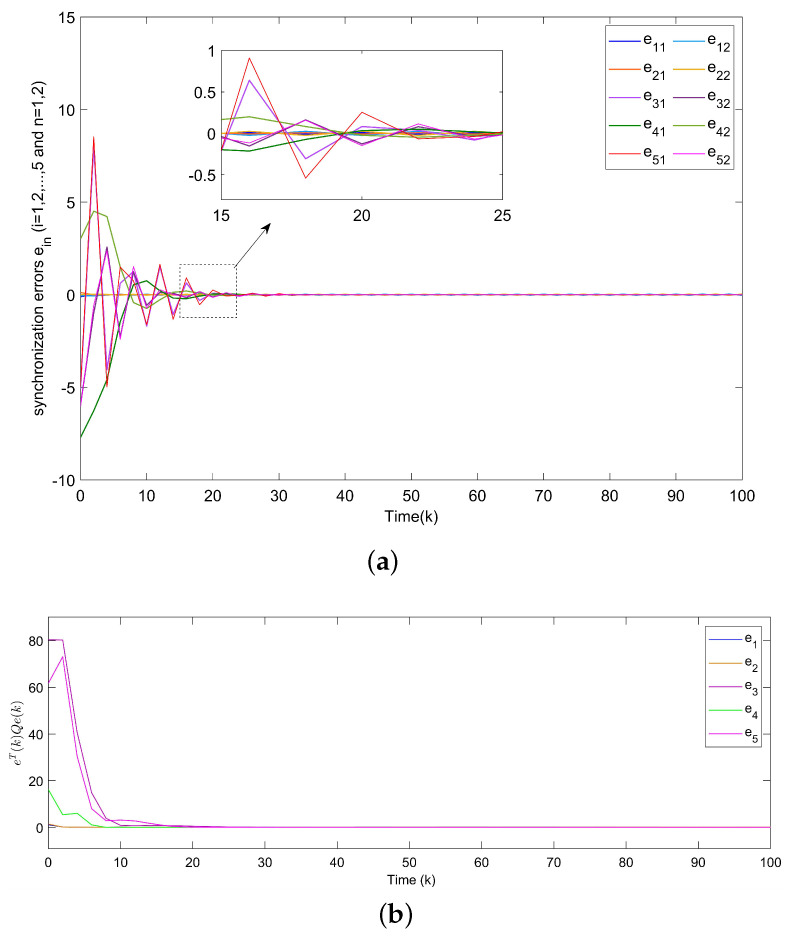
(**a**) Synchronization errors $e_{in}$ of closed-loop TSFDCNs with controllers. (**b**) Curves of Lyapunov terms $e_{i}^{T}\left(k\right)Q_{i}e_{i}\left(k\right)$.

**Figure 5:**
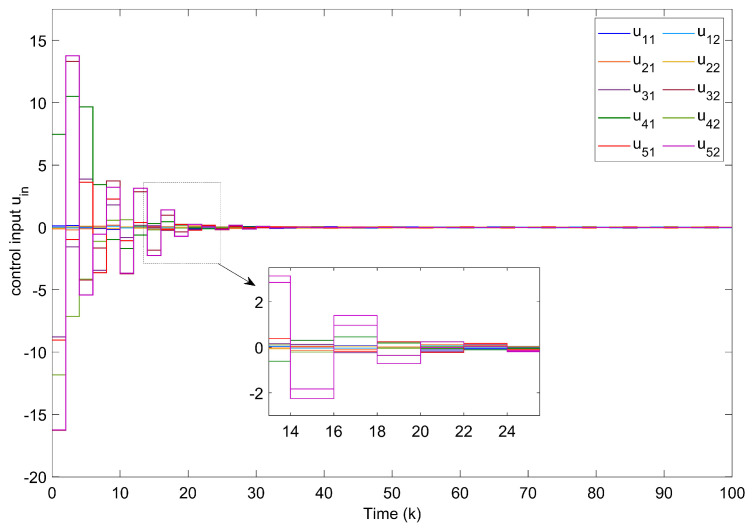
Curves of control inputs.

For Example 1, the initial states of nodes are selected as $x_{1}\left(k\right)={\left(-2.4,-0.9\right)}^{T}$, $x_{2}\left(k\right)={\left(2,-1.5\right)}^{T}$, $x_{3}\left(k\right)={\left(-2.2,3.3\right)}^{T}$, $x_{4}\left(k\right)={\left(1.6,-1.8\right)}^{T}$, $x_{5}\left(k\right)={\left(-2.8,3.5\right)}^{T}$, and $s\left(k\right)={\left(2,-1\right)}^{T}$ for $k\in \left\{-3\right.,\left.-2,-1,0\right\}$. Shown in Figure 4a, with controllers, the closed-loop error system of TSFDCNs gradually converges to stability in finite-time. Besides, Figure 4b displays the convergence performance of Lyapunov term $e_{i}^{T}\left(k\right)Q_{i}e_{i}\left(k\right)$ and proposed stability theory is further verified. Figure 5 shows the trajectory of control inputs. Compared with open-loop results, controlled networks can synchronize to the isolated node.

The selection of parameter values affects the synchronization control performance of TSFDCNs. According to Theorem 1, the bounds of $m_{2}$ are restrained by the upper bound of the time delay. Assume that $\tau_{m}=1$ and other parameters are set as the same as in previous experiment. In Table 1, the allowable minimum values of $m_{2}$ for different $\tau_{M}$ are solved from the presented conditions in Theorem 1, which indicates that $m_{2}$ increases with the rise of $\tau_{M}$.

Notice that there exist two special issues with the change of parameters $\sigma_{i}$ and $\pi_{i}$. When $\sigma_{i}=0$, we obtain the static event-triggered condition used in [18]:

$$k_{s+1}^{i}=\inf\left\{k\in N\left|k>k_{s}^{i},\;\right.\varepsilon_{i}^{T}\left(k\right)\Omega_{i}\varepsilon_{i}\left(k\right)\right.-\pi_{i}e_{i}^{T}\left(k\right)\Omega_{i}e_{i}\left(k\right)>\left.0\right\}.$$

When $\sigma_{i}=\pi_{i}=0$, the condition is reduced as with the periodic triggered case proposed in [39],

$$k_{s+1}^{i}=\inf\left\{k\in N\left|k>k_{s}^{i},\;\right.\varepsilon_{i}^{T}\left(k\right)\Omega_{i}\varepsilon_{i}\left(k\right)\right.>\left.0\right\}$$

**Figure 6:**
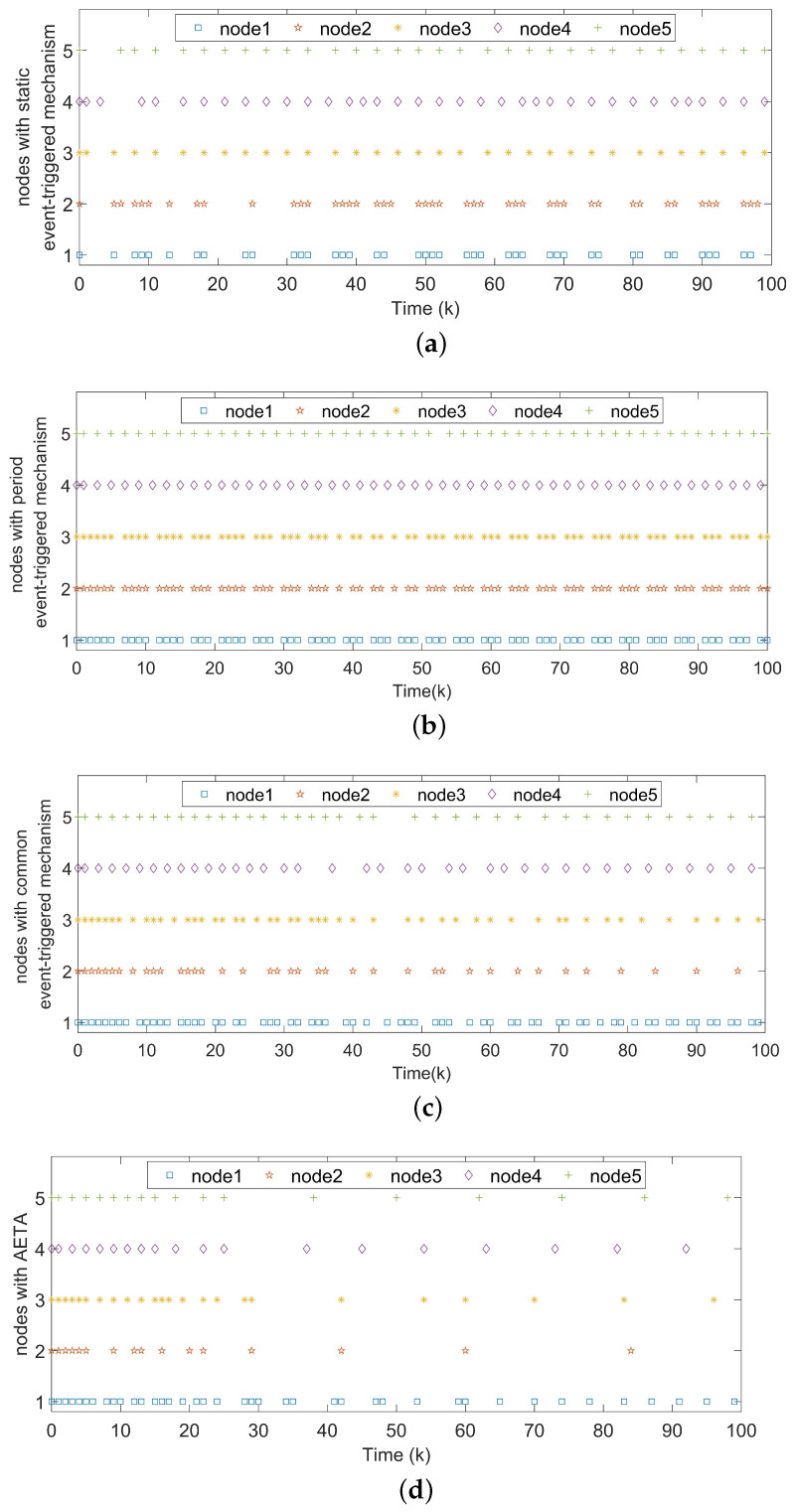
(**a**) Triggered instants under the static event-triggered mechanism in [18]. (**b**) Triggered instants under the periodic event-triggered mechanism in [39]. (**c**) Triggered instants under the static event-triggered mechanism in [48]. (**d**) Triggered instants under the AETA.

**Figure 7:**
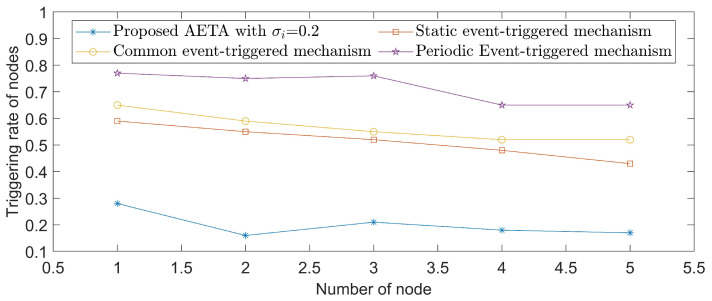
The triggering rates of AETA and methods in [18,39,48] for various nodes.

**Figure 8:**
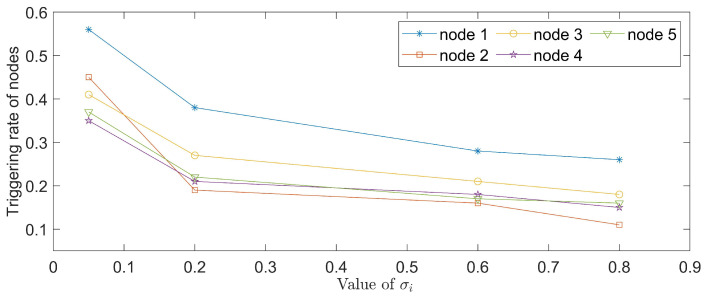
The triggering rates of five nodes for varying $\sigma_{i}$.

With hope to evaluate the performance, a set of experiments is conducted among four event-triggered approaches. The corresponding results are displayed in Figure 6, where Figure 6a shows the corresponding static triggered case in [18], Figure 6b shows the periodic triggered case in [39], Figure 6c shows the event-triggered method in [48] and the last one represents the performance of our proposed AETA with $\sigma_{i}=0.6$. It is obvious that the triggered times in Figure 6d are far fewer than in the other three cases. The triggering rates of five nodes under different mechanisms are further shown in Figure 7, where parameter $\sigma_{i}$ is set as 0.2 and AETA is obviously superior to other methods. Based on the triggering condition (7), the triggering rate is greatly influenced by the selection of $\sigma_{i}$. Then, the relationship between triggering rate and varying values of $\sigma_{i}$ are provided in Figure 8.

**Remark** **11.** *To quantize results, Table 2 is given to show the average triggering rate (ATR) of network nodes under several existing methods and different values of $\sigma_{i}$ in AETA. With respect to the index of ATR, AETA outperforms the methods in [18,39,48]. Moreover, the ATR increases gradually when the value of $\sigma_{i}$ decreases to zero, which is also clearly reflected in Figure 8. In conclusion, the communication burden of the control process is effectively saved by AETA, compared with other event-triggered methods.*

**Figure 9:**
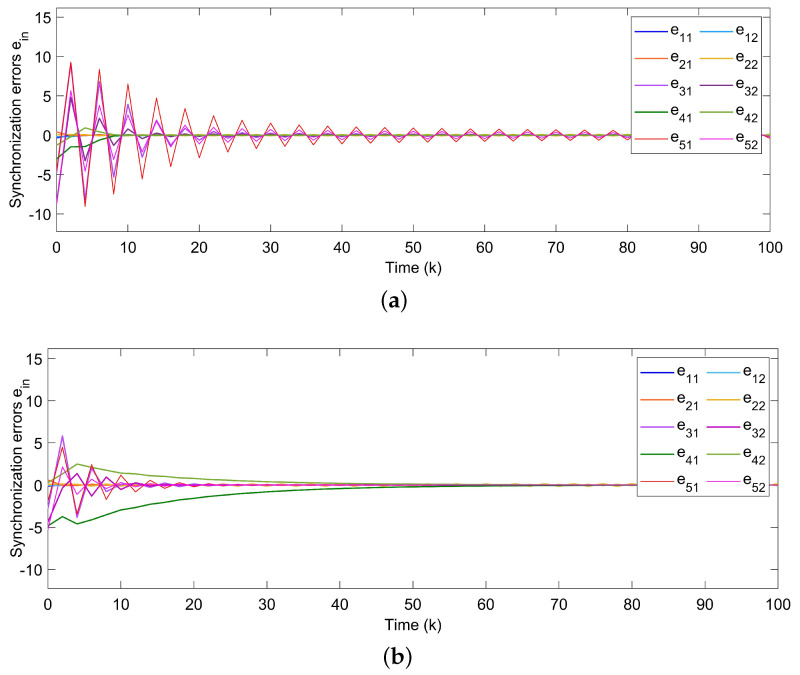
(**a**) Synchronization errors by Theorem 2 in [29]. (**b**) Synchronization errors by Theorem 2 in [44].

Since system parameters were set in the last subsection, we introduce the method in [29,44] to compare system performance and related simulation results are given in Figure 9. As shown in Figure 9a, by Theorem 2 in [29], the errors of the closed-loop system cannot reach the synchronized state in the setting time. By Theorem 2 in [44], displayed in Figure 9b, synchronization errors can converge to zero when *k* gets near 50, while the optimal convergence time is k=26 with the proposed controller in this paper. It reveals that our approach has a superior synchronization performance.

In order to further verify the usefulness of our proposed strategy in a practical system, the following example will introduce a discrete-time chaotic network to achieve the finite-time synchronization.

**Example** **2.** *Consider the TSFDCNs containing three nodes and each node is regarded as a chaotic subsystem, where $x_{i}\left(k\right)={\left(x_{i1}\left(k\right),x_{i2}\left(k\right)\right)}^{T}$, i=1, 2, 3. Choosing fuzzy membership functions η1(θ(k))=(1−sin2(k))(1−sin2(k))22 and η2(θ(k))=(1+sin2(k))(1+sin2(k))22 for two T-S fuzzy rules, some parameter matrices are defined as follows:*

*$A_{1}=\left[\begin{array}{cc} 0.89 & 0\\ 0 & 0.91 \end{array}\right]$, $A_{2}=\left[\begin{array}{cc} 0.9 & 0\\ 0 & 0.9 \end{array}\right]$, $B_{11}=\left[\begin{array}{cc} 0.21 & -0.012\\ -1.51 & 0.32 \end{array}\right]$, *

*$B_{21}=\left[\begin{array}{cc} 0.18 & -0.011\\ -1.6 & 0.32 \end{array}\right]$, $B_{12}=\left[\begin{array}{cc} -0.15 & -0.01\\ 0.012 & -0.14 \end{array}\right]$, $B_{22}=\left[\begin{array}{cc} -0.16 & -0.01\\ 0.015 & -0.12 \end{array}\right]$.*

*The node activation functions are given as:*

*$f\left(x_{i}\left(k\right)\right)=\left[\begin{array}{c} \tanh(x_{i1}\left(k\right))\\ \tanh(x_{i2}\left(k\right)) \end{array}\right]$, $h\left(x_{i}\left(\Delta_{\tau }\right)\right)=\left[\begin{array}{c} \tanh(x_{i1}\left(\Delta_{k}\right))\\ \tanh(x_{i2}\left(\Delta_{k}\right)) \end{array}\right]$.*

**Figure 10:**
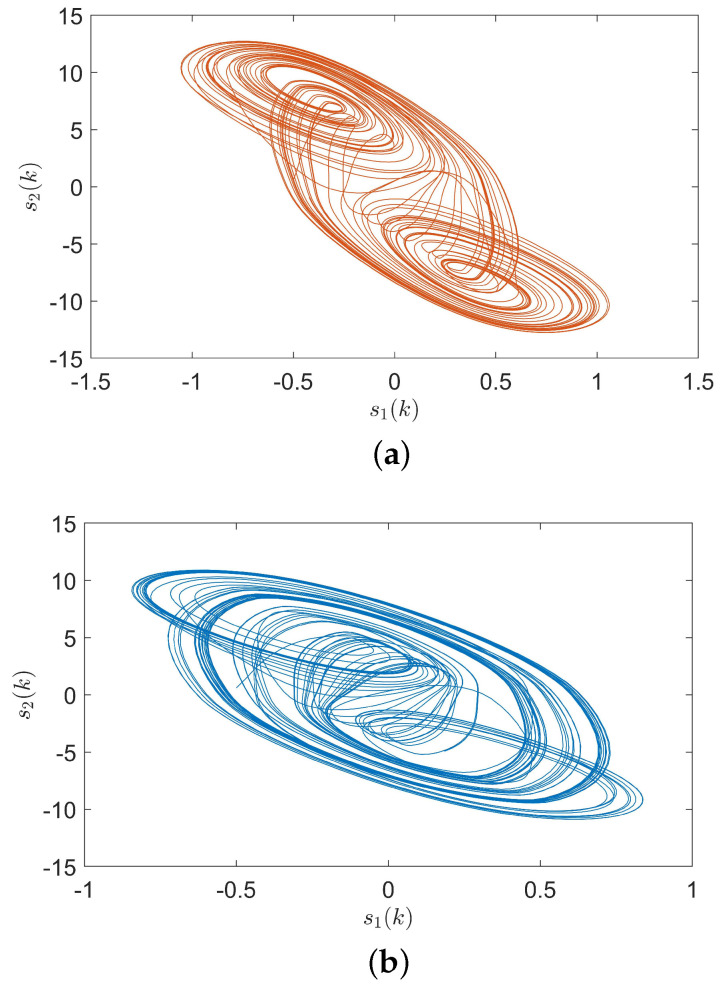
Chaotic trajectories of two fuzzy modes with initial condition $\tilde{x}\left(k\right)={\left(-0.5,0.6\right)}^{T}$. (**a**) Rule 1. (**b**) Rule 2.

*The time-varying delay for all network nodes is set as τ(k)=e0.1ke0.1k0.1(1+e0.1k)0.1(1+e0.1k), with $\tau_{m}=5$ and $\tau_{M}=10$. The network system also suffers from disturbance νi(k)=0.5e−0.1ksin(πkπk22). In Figure 10, the chaotic trajectories for two fuzzy modes are demonstrated clearly under the initial condition $s\left(k\right)={\left(-0.5,0.6\right)}^{T}$ for $k\in {\left[-25,0\right]}_{Z}$. In addition, let c=0.9, Γ=I and the undirected coupled configuration matrices for two rules as:*

$$G_{1}=\left[\begin{array}{ccc} -0.3 & 0.1 & 0.2\\ 0.3 & -0.4 & 0.1\\ 0.2 & 0.1 & -0.3 \end{array}\right],\;G_{1}=\left[\begin{array}{ccc} -0.2 & 0.1 & 0.1\\ 0.2 & -0.4 & 0.2\\ 0.1 & 0.2 & -0.3 \end{array}\right]\;$$

*Some system parameters are defined as Φ=I, $m_{1}=1.5$, $m_{2}=15$, $T_{m}=50$, $\Omega_{i}=I$, $\pi_{i}=0.2$, $\sigma_{i}=0.65$, ${ƛ}_{i}=1.5$, $d_{i0}=0.1$ and $\tilde{w}=0.5$. Suppose that node 1 and node 3 are controlled by synchronization conditions in Theorem 1, we can then obtain the fuzzy controller gains $\Pi_{li}$ as follows:*

*$\Pi_{11}=\left[\begin{array}{cc} 1.3589 & -0.0046\\ -0.0046 & -1.3381 \end{array}\right]$, $\Pi_{12}=\left[\begin{array}{cc} 1.4106 & -0.0057\\ -0.0057 & -1.3699 \end{array}\right]$,*

*$\Pi_{31}=\left[\begin{array}{cc} 0.9526 & -0.0052\\ -0.0052 & 0.9176 \end{array}\right]$, $\Pi_{32}=\left[\begin{array}{cc} 1.0817 & -0.0105\\ -0.0105 & 0.9630 \end{array}\right]$.*

**Figure 11:**
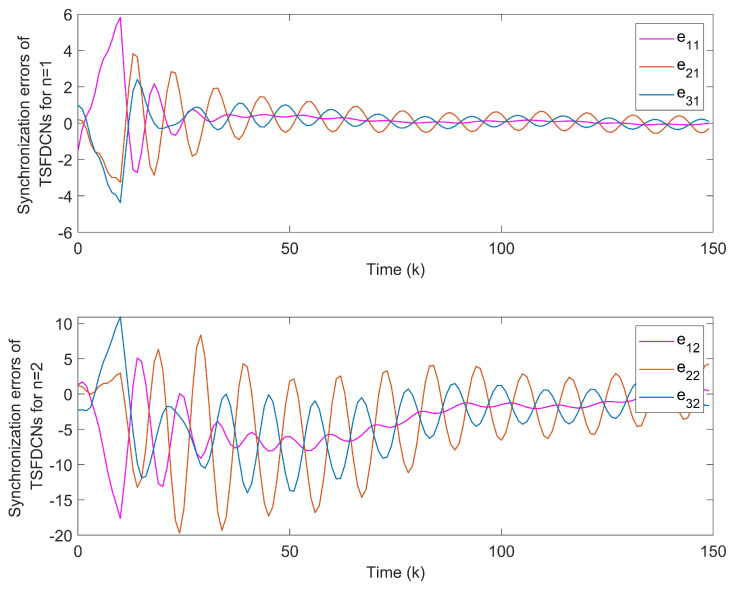
Synchronization errors of chaotic TSFDCNs without control.

**Figure 12:**
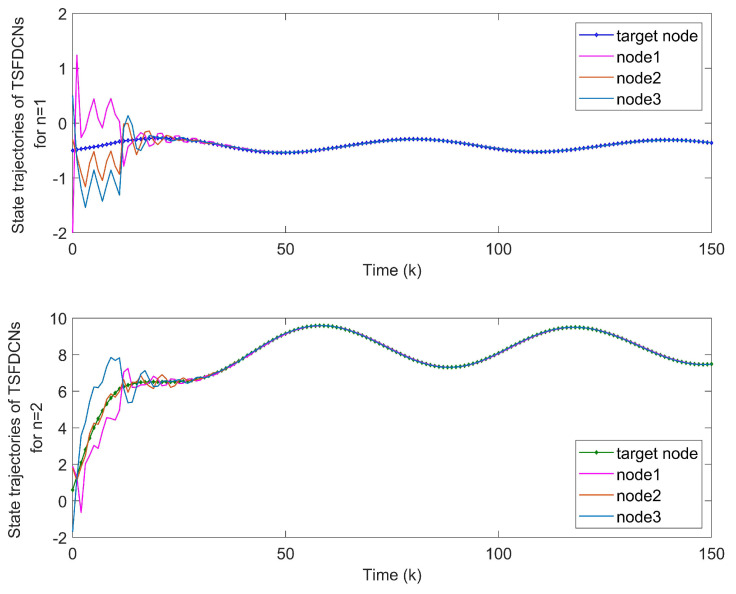
State trajectories of network nodes in chaotic TSFDCNs.

**Figure 13:**
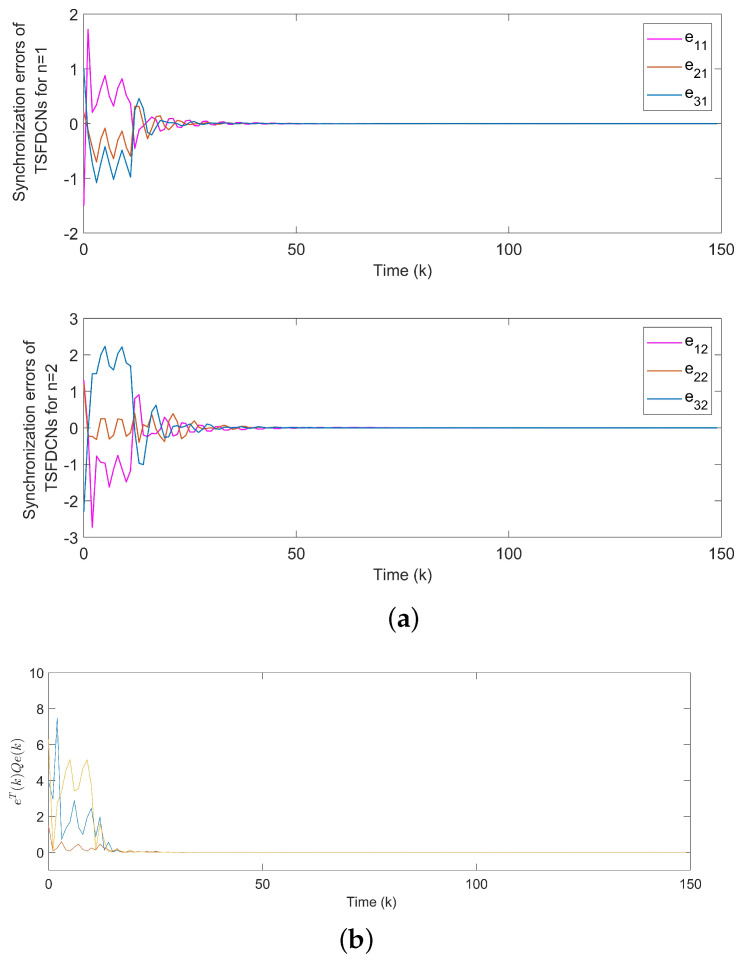
(**a**) Synchronization errors of chaotic TSFDCNs under control. (**b**) Curves of Lyapunov terms $e_{i}^{T}\left(k\right)Q_{i}e_{i}\left(k\right)$.

**Figure 14:**
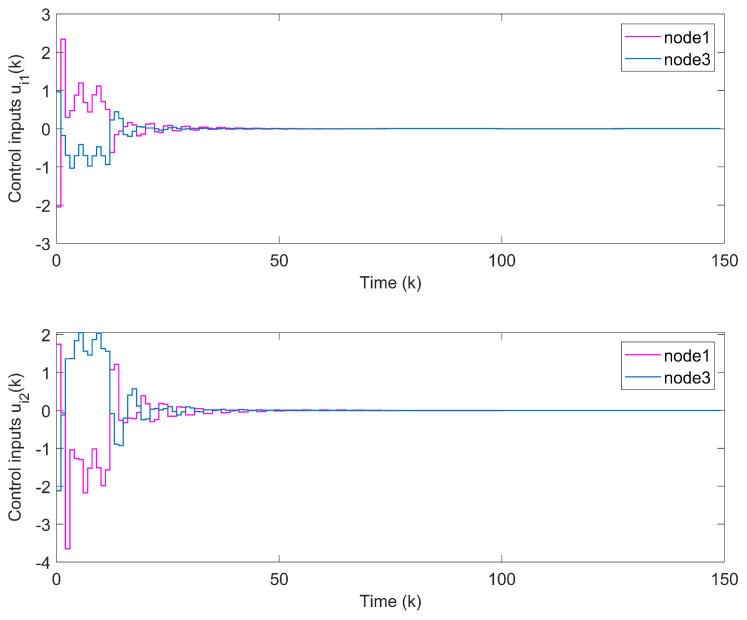
Curves of control inputs.

**Figure 15:**
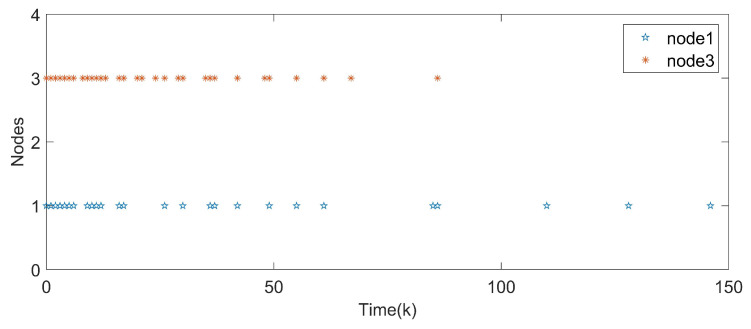
Triggered instants of pinned nodes.

**Figure 16:**
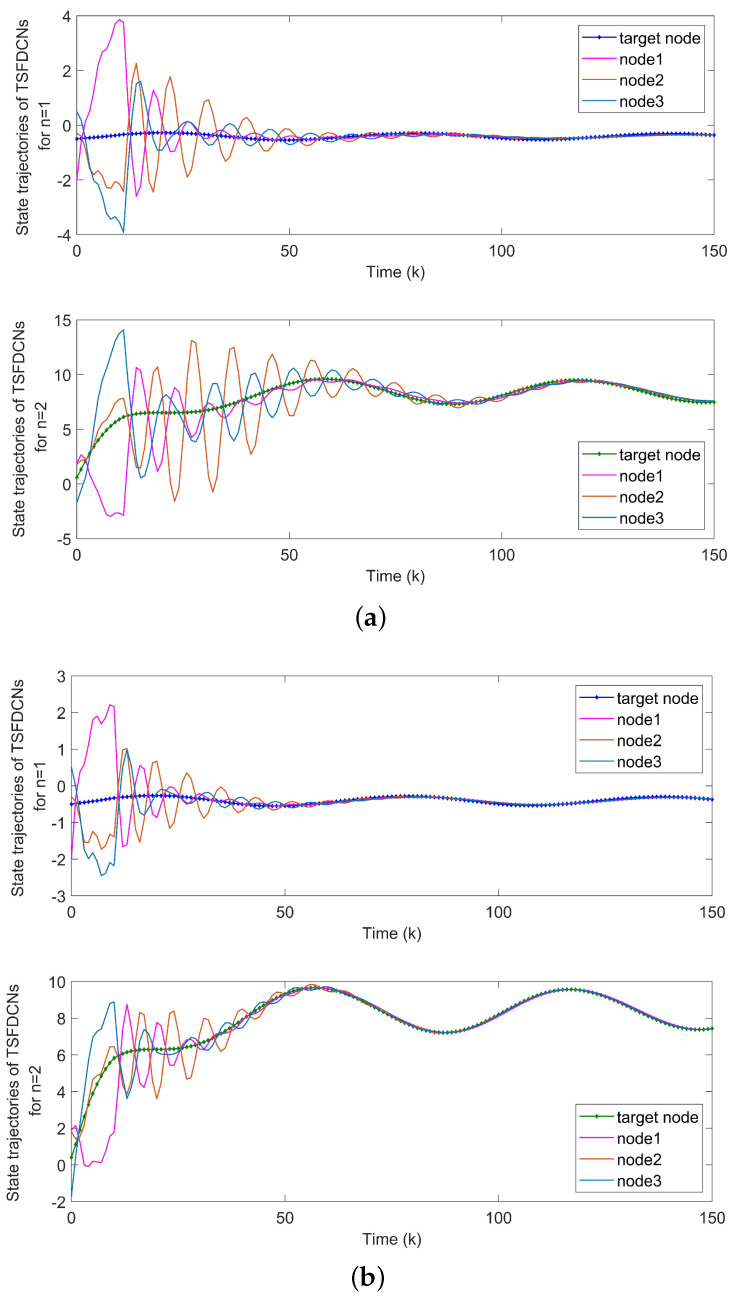
Performance of two existing methods. (**a**) State trajectories of network nodes by Theorem 2 in [29]. (**b**) State trajectories of network nodes by Theorem 3.1 in [34].

*With the initial values $x_{1}\left(k\right)={\left(-1,0.6\right)}^{T}$, $x_{2}\left(k\right)={\left(-0.3,0.8\right)}^{T}$ and $x_{3}\left(k\right)={\left(0.5,-0.7\right)}^{T}$, synchronization error curves of open-looped TSFDCNs are shown in Figure 11. Through introducing the control signals to nodes, the state trajectory of the target node can be tracked well by three network nodes and synchronization errors can converge in finite time, which are exhibited via Figure 12 and Figure 13. In Figure 14, the corresponding control inputs are drawn. The triggered instants of controlled nodes are given by Figure 15, where ATR is calculated as 19%. On the basis of this chaotic system, we compare the results of two existing synchronous control techniques and show them in Figure 16. Intuitively, by these two methods, the state trajectory is unable to be tracked within k=50 and oscillations are bigger. The specific convergence time is listed in Table 3; it implies that the method proposed in Theorem 1 outperforms the other two.*

By means of Theorem 2, the finite-time synchronization of DCNs can be achieved, which will be proved by the following example.

**Example** **3.** *Consider the DCNs including four nodes (N = 4) with the following parameters:*

*$A=-I_{3}$, $B_{1}=\left[\begin{array}{ccc} -0.2 & 0.5 & 0.4\\ 0.3 & -0.6 & 0.1\\ 0.3 & 0.2 & -0.5 \end{array}\right]$, $B_{2}=\left[\begin{array}{ccc} -0.3 & 0.2 & 0.1\\ 0.2 & -0.1 & 0.3\\ 0.4 & 0.1 & -0.2. \end{array}\right]$*

*The nonlinear activation functions* f(·) *and* h(·) *are:*

$$f\left(x_{i}\left(k\right)\right)=\left[\begin{array}{c} 0.4x_{1}\left(k\right)-\tanh\left(0.3x_{1}\left(k\right)\right)\\ \begin{array}{c} 0.3x_{2}\left(k\right)-\tanh\left(-0.4x_{2}\left(k\right)\right)\\ 0.5x_{3}\left(k\right)-\tanh\left(0.5x_{1}\left(k\right)\right) \end{array} \end{array}\right]$$

$$h\left(x_{i}\left(\Delta_{\tau }\right)\right)=\left[\begin{array}{c} 0.3x_{1}\left(\Delta_{\tau }\right)-0.1\tanh\left(0.1x_{1}\left(\Delta_{\tau }\right)\right)\\ \begin{array}{c} -0.2x_{2}\left(\Delta_{\tau }\right)+0.3\tanh\left(0.3x_{2}\left(\Delta_{\tau }\right)\right)\\ 0.1x_{3}\left(\Delta_{\tau }\right)+0.2\tanh\left(-0.2x_{2}\left(\Delta_{\tau }\right)\right). \end{array} \end{array}\right]$$

**Figure 17:**
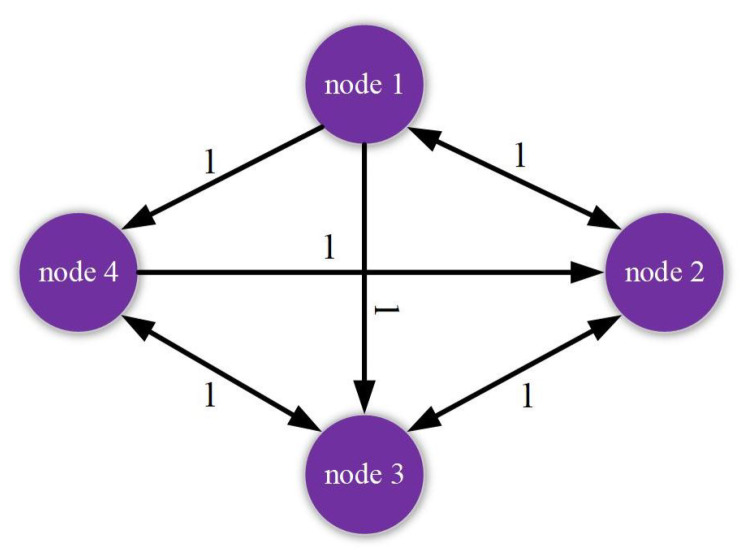
Communication structure of coupled nodes in DCNs.

*Let τ=2, c=0.8, $\Gamma =-0.6I_{3}$, and the topological structure in Figure 17 defines the coupled configuration matrix as:*

$$G=\left[\begin{array}{cccc} -2 & 1 & 1 & 1\\ 1 & -3 & 1 & 0\\ 0 & 1 & -2 & 1\\ 0 & 1 & 1 & -2. \end{array}\right]$$

In simulations, we choose Φ=I, $m_{1}=0.1$, $m_{2}=3$, $\pi_{i}=0.15$, $\sigma_{i}=0.8$, ƛ=1.2, $d_{i0}=0$, $\hbar^{*}=1$, $\hbar_{1}=\hbar_{2}=0.8$, and the initial system states are assumed as $x_{1}\left(k\right)={\left(-0.2,1.1,-0.5\right)}^{T}$, $x_{2}\left(k\right)={\left(-2.5,-1.8,0.2\right)}^{T}$, $x_{3}\left(k\right)={\left(-0.9,-2.8,1\right)}^{T}$, $x_{4}\left(k\right)=(0.5,$ ${-1.8,0.1)}^{T}$, $s\left(k\right)={\left(1,-1,2\right)}^{T}$ for k=−2. We deduce the following control gains:

$$\Pi_{1}=\left[\begin{array}{ccc} -10.8652 & 1.0237 & -2.6701\\ 0 & 5.2051 & -2.0032\\ -5.0721 & -8.2006 & 5.1233 \end{array}\right],\;\Pi_{2}=\left[\begin{array}{ccc} -10.5406 & 1.4589 & -2.8723\\ 0 & 5.2611 & -2.0742\\ -5.1290 & -8.2107 & 5.2118 \end{array}\right]$$

$$\Pi_{3}=\left[\begin{array}{ccc} -10.6315 & 1.2790 & -2.2130\\ 0 & 5.1843 & -2.0637\\ -5.3428 & -8.1592 & 5.1341 \end{array}\right],\;\Pi_{4}=\left[\begin{array}{ccc} -10.5893 & 1.3122 & -2.4685\\ 0 & 5.2417 & -2.1090\\ -5.1691 & -8.2502 & 5.2782 \end{array}\right].$$

**Figure 18:**
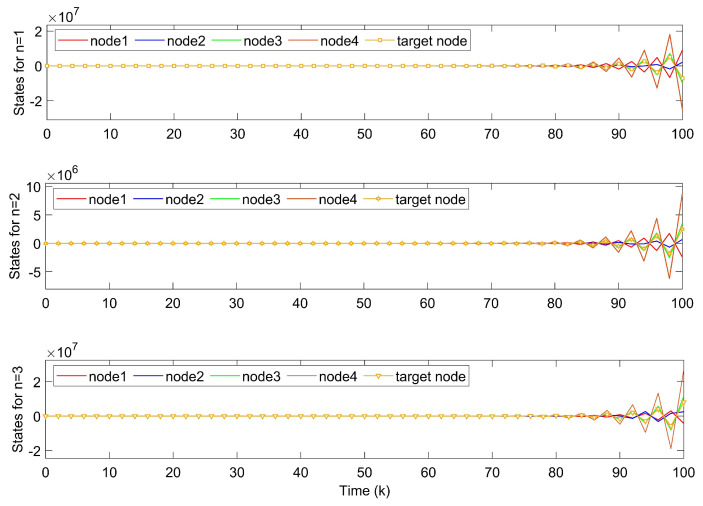
States of nodes $x_{i1},\;x_{i2},\;x_{i3}$ in DCNs.

**Figure 19:**
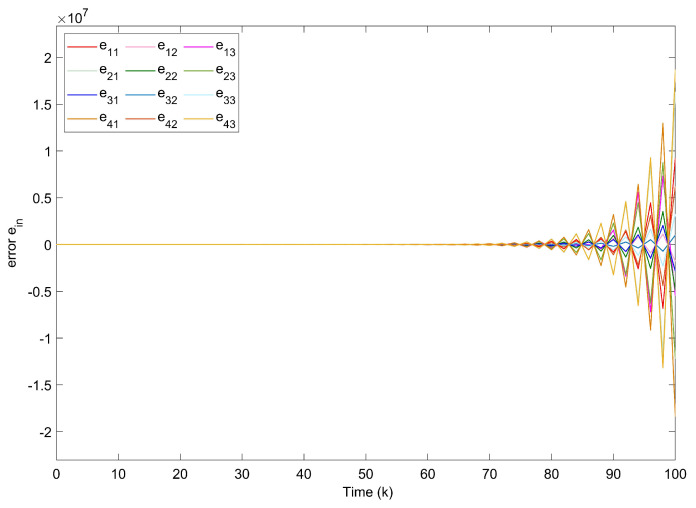
Synchronization errors $e_{in}$ without controllers of DCNs.

**Figure 20:**
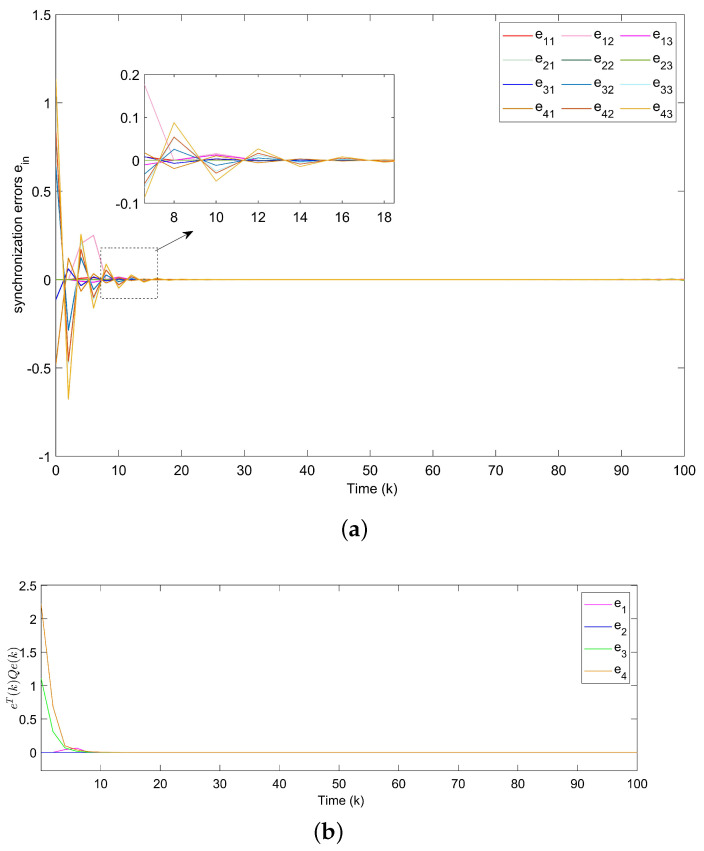
(**a**) Synchronization errors $e_{in}$ of closed-loop DCNs with controllers. (**b**) Curves of Lyapunov terms $e_{i}^{T}\left(k\right)Q_{i}e_{i}\left(k\right)$.

**Figure 21:**
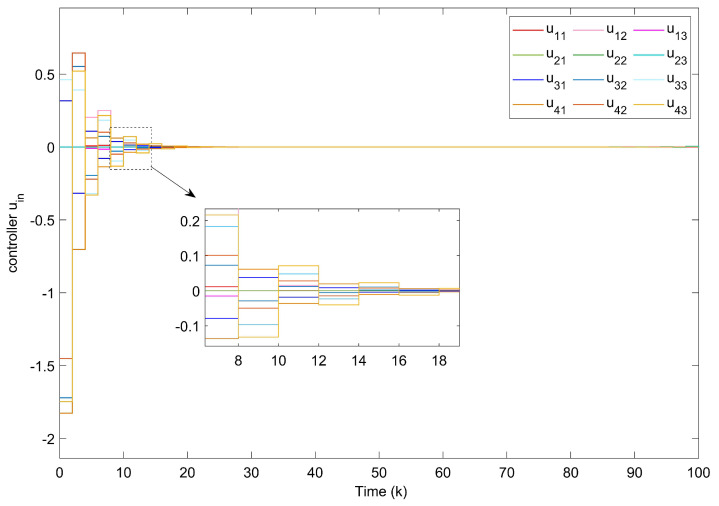
Curves of control inputs.

**Figure 22:**
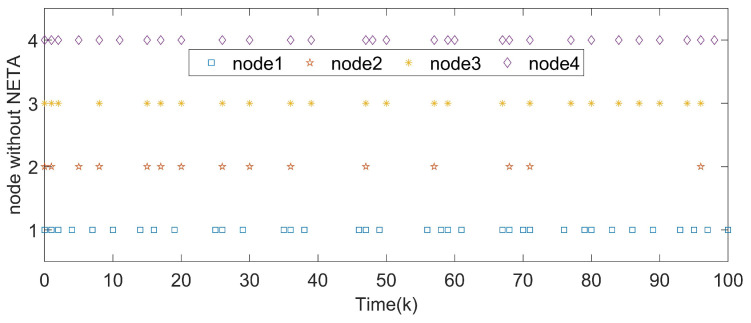
Triggered instants of pinned nodes in DCNs.

*The states of nodes in DCNs are indicated in Figure 18. From Figure 19, we get the synchronization errors which diffuse with time mainly due to coupling effects and delays. Figure 20a indicates that states of DCNs can be ultimately finite-time synchronized, where the minimum $T_{m}$ is computed as 19. Lyapunov stability is obviously obtained by Figure 20b, where curves of $e_{i}^{T}\left(k\right)Q_{i}e_{i}\left(k\right)$ are plotted. Particularly, using the model in Example 3, Table 4 provides the optimal finite time $T_{m}$ for various $m_{2}$. It is obvious that the enlargement of $m_{2}$ results in a longer minimum convergence time. In Figure 21, the performance of the controller is displayed. The triggered instants of DCNs are depicted in Figure 22 and ATR is 25.67%. As a result, the effectiveness of the proposed theory and method is proved.*

## 5. Conclusions

In this paper, the finite-time pinning synchronization control problem has been studied for TSFDCNs with time-varying delays. By means of the T-S fuzzy model, the dynamical behaviors of more general delayed DCNs with couplings and external disturbance are analyzed. In order to further reduce the communication burden of the control update, a discrete AETA is introduced with an adaptive threshold to the controller design, and the triggering rate can be obviously decreased in the system examples. Based on finite-time Lyapunov–Krasovskii functionals, sufficient synchronization criteria are derived to guarantee the finite-time stability of the closed-loop error system. By considering LMI constraints related to an optimization algorithm for minimum finite time, the desired gains of the fuzzy pinning controller are further obtained. The effectiveness and advantages of our proposed control strategy are proved by several experiments, where synchronization errors are converged with a shorter time in comparison. However, computation complexity rises with the number of nodes and needs to be reduced, which will be appreciated in the following study. For a future research topic, the proposed method will be extended to study control strategies of TSFDCNs subject to different disturbances or cyber-attacks, as well as to analyze the finite-time synchronization of Markov DCNs.

## Figures and Tables

**Table 1:** The allowable minimum values of $m_{2}$ for different $\tau_{M}$.

$\tau_{M}$	2	3	4	5	6
$m_{2}$	152.6436	156.5210	163.4011	175.2630	198.8712

**Table 2:** Comparison of triggering rates in different cases.

Method	Node 1	Node 2	Node 3	Node 4	Node 5	ATR
$\sigma_{i}=0.8$	26%	11%	18%	15%	16%	17.20%
$\sigma_{i}=0.6$	28%	16%	21%	18%	17%	20%
$\sigma_{i}=0.2$	38%	19%	27%	21%	22%	25.40%
$\sigma_{i}=0.05$	56%	45%	41%	35%	37%	42.80%
Static event-triggered mechanism in [18]	59%	55%	52%	48%	43%	51.40%
Common event-triggered mechanism in [48]	65%	59%	55%	52%	52%	56.60%
Periodic Event-triggered mechanism in [39]	77%	75%	76%	65%	65%	71.60%

**Table 3:** Comparison of convergence time $T_{c}$.

Method	Theorem 1	Theorem 2 in [29]	Theorem 3.1 in [34]
** $T_{c}/k$ **	43	>150	87

**Table 4:** Calculated minimum $T_{m}$ for various values of $m_{2}$.

$m_{2}$	2	3	5	10	15	20
$T_{m}/k$	17	19	23	28	35	48

## Data Availability

Not applicable.

## Abbreviations

The following abbreviations are used in this manuscript:

DCNs	Discrete complex networks
TSFDCNs	T-S fuzzy discrete complex networks
AETA	Adaptive event-triggered approach
LMIs	Linear matrix inequalities

## Appendix A. Proof of Theorem 1

Choose the following Lyapunov-Krasovskii functional candidate for the error system (11):

(A1) $$V\left(k\right)=\sum_{q=1}^{5}V_{q}\left(k\right)$$

where

$V_{1}\left(k\right)=e^{T}\left(k\right)Qe\left(k\right),$

$V_{2}\left(k\right)=\sum_{i=1}^{N}y\sigma_{i}d_{i}\left(k\right)$

$V_{3}\left(k\right)=\sum_{i=\Delta_{k}}^{k-1}y^{i-k+1}e^{T}\left(i\right)\Upsilon_{1}e\left(i\right)+\sum_{j=-\tau_{M}+2}^{-\tau_{m}+1}\sum_{i=k+s-1}^{k-1}y^{i-k+1}e^{T}\left(i\right)\Upsilon_{1}e\left(i\right),$

$V_{4}\left(k\right)=\sum_{j=-\tau_{M}}^{-\tau_{m}-1}\sum_{℘=j}^{-1}\sum_{i=k+℘}^{k-1}y^{i-k+1}\beta^{T}\left(i\right)\Upsilon_{2}\beta \left(i\right)$

$V_{5}\left(k\right)=\left(\tau_{M}-\tau_{m}\right)\sum_{j=-\tau_{M}}^{-\tau_{m}-1}\sum_{i=k+j}^{k-1}y^{i-k+1}\beta^{T}\left(i\right)\Upsilon_{3}\beta \left(i\right)+\tau_{m}\sum_{j=-\tau_{m}}^{-1}\sum_{i=k+j}^{k-1}y^{i-k+1}\beta^{T}\left(i\right)\Upsilon_{4}\beta \left(i\right)\;$ and β(i)=e(i+1)−e(i). For simplicity, let

$\gamma^{T}\left(k\right)=\left[{\tilde{\gamma }}^{T}\left(k\right),\tilde{d}\left(k\right),\varepsilon^{T}\left(k\right)\right],$

$\begin{array}{c} {\tilde{\gamma }}^{T}\left(k\right)=[e^{T}\left(k\right),e^{T}\left(\Delta_{k}\right),e^{T}\left(\Delta_{M}\right),e^{T}\left(\Delta_{m}\right),\kappa_{1}^{T},\kappa_{2}^{T},\kappa_{3}^{T},\kappa_{4}^{T},\kappa_{5}^{T},\kappa_{6}^{T},\\ \;\;\;\;\;\;\;\;\;\;\;\;\;\;\;\;\;\;\;\;\;\;F^{T}\left(k\right),H^{T}\left(\Delta_{k}\right),w^{T}\left(k\right)],\;\;\;\;\Delta_{M}=k-\tau_{M},\;\;\Delta_{m}=k-\tau_{m}, \end{array}$

$\tilde{d}\left(k\right)=diag\left\{d_{1}^{1/2}\left(k\right),d_{2}^{1/2}\left(k\right),\ldots ,d_{N}^{1/2}\left(k\right)\right\},$

$\kappa_{1}=\frac{1}{\tau_{m}+1}\sum_{i=\Delta_{m}}^{k}e(i)$ *,* $\kappa_{2}=\frac{1}{\tau \left(k\right)-\tau_{m}+1}\sum_{i=\Delta_{k}}^{\Delta_{m}}e(i)$ *,* $\kappa_{3}=\frac{1}{\tau_{M}-\tau \left(k\right)+1}\sum_{i=\Delta_{M}}^{\Delta_{k}}e(i),$

$\kappa_{4}=\frac{2}{\left(\tau_{m}+1\right)\left(\tau_{m}+2\right)}\sum_{j=-\tau_{m}}^{0}\sum_{i=k+j}^{k}e(i)$ *,* $\kappa_{5}=\frac{2}{\left(\tau \left(k\right)-\tau_{m}+1\right)\left(\tau \left(k\right)-\tau_{m}+2\right)}\sum_{j=-\tau (k)}^{-\tau_{m}}\sum_{i=k+j}^{\Delta_{m}}e(i),$

$\kappa_{6}=\frac{2}{\left(\tau_{M}-\tau \left(k\right)+1\right)\left(\tau_{M}-\tau \left(k\right)+2\right)}\sum_{j=-\tau_{M}}^{-\tau (k)}\sum_{i=k+j}^{\Delta_{k}}e(i)$

(A2)
$$\gamma_{2}^{T}\left(k\right)=\left[F^{T}\left(k\right),H^{T}\left(\Delta_{k}\right)\right],\gamma_{3}^{T}\left(k\right)=\left[w^{T}\left(k\right),d^{1/2}\left(k\right),\varepsilon^{T}\left(k\right)\right].$$

For $k\in \left[k_{s}^{i},k_{s+1}^{i}\right)$, taking the forward difference of $V_{q}\left(k\right)$, we have

(A3) $$\begin{array}{c} \Delta V_{1}\left(k\right)=V_{1}\left(k+1\right)-V_{1}\left(k\right)\\ \;\;\;\;\;\;\;\;\;\;\;\;\;\;\;\;\;\;\;\;\;\;\;\;\;\;\;=e^{T}\left(k+1\right)Qe\left(k+1\right)-y^{-1}e^{T}\left(k\right)Qe\left(k\right)+\left(y^{-1}-1\right)V_{1}\left(k\right)\\ \;\;\;\;\;\;\;\;\;\;\;\;\;\;\;\;\;\;\;\;\;\;\;\;\;\;\;\;=\beta \left(k\right)Q\;\beta \left(k\right)+2e^{T}\left(k\right)Qe\left(k+1\right)-e^{T}\left(k\right)Qe\left(k\right)\\ \;\;\;\;\;\;\;\;\;\;\;\;\;\;\;\;\;\;\;\;\;\;\;\;\;\;\;\;\;\;\;\;\;\;\;-y^{-1}e^{T}\left(k\right)Qe\left(k\right)+\left(y^{-1}-1\right)V_{1}\left(k\right). \end{array}$$

(A4) $$\begin{array}{c} \Delta V_{2}\left(k\right)=V_{2}\left(k+1\right)-V_{2}\left(k\right)\\ \;\;\;\;\;\;\;\;\;\;\;\;\;\;\;\;\;\;\;\;\;\;\;\;\;\;\;\;=\sum_{i=1}^{N}\sigma_{i}\left[\left(\frac{y}{\;{ƛ}_{i}}-1\right)d_{i}\left(k\right)+y\pi_{i}e_{i}^{T}\left(k\right)\Omega_{i}e_{i}\left(k\right)\right.\left.-y\varepsilon_{i}^{T}\left(k\right)\Omega_{i}\varepsilon_{i}\left(k\right)\right]+\left(y^{-1}-1\right)V_{2}\left(k\right)\\ \;\;\;\;\;\;\;\;\;\;\;\;\;\;\;\;\;\;\;\;\;\;\;\;\;\;\;\;\;=\sum_{i-1}^{N}\sigma_{i}\left[\left(\frac{y}{\;{ƛ}_{i}}-1\right)d_{i}\left(k\right)+yE_{i}^{T}{\tilde{\Omega }}_{i}E_{i}\right]+\left(y^{-1}-1\right)V_{2}\left(k\right). \end{array}$$

where $E_{i}^{T}=\left[e_{i}^{T}\left(k\right),\varepsilon_{i}^{T}\left(k\right)\right]$, ${\tilde{\Omega }}_{i}=diag\left\{\pi_{i}\Omega_{i},-\Omega_{i}\right\}$. According to the adaptive event-triggered condition (7), it yields

(A5) $$\sum_{i=1}^{N}\left[\sigma_{i}d_{i}\left(k\right)+\pi_{i}e^{T}\left(k\right)\Omega_{i}e_{i}\left(k\right)-\varepsilon_{i}^{T}\left(k\right)\Omega_{i}\varepsilon_{i}\left(k\right)\right]\geq 0$$

which means for $\hbar^{*}>0$

(A6) $$\hbar^{*}\sum_{i=1}^{r}\sigma_{i}\left[d_{i}\left(k\right)+E_{i}^{T}\Omega E_{i}\right]⩾0.$$

(A7) $$\begin{array}{c} \Delta V_{3}\left(k\right)=V_{3}\left(k+1\right)-V_{3}\left(k\right)\\ \;\;\;\;\;\;\;\;\;\;\;\;\;\;\;\;\;\;\;\;\;\;\;\;\;\;\;\;\;=e^{T}\left(k\right)\Upsilon_{1}e\left(k\right)+\sum_{i=\Delta_{k+1}}^{k-1}y^{i-k}e^{T}\left(i\right)\Upsilon_{1}e\left(i\right)+\sum_{j=-\tau_{M}+2}^{-\tau_{m}+1}\sum_{i=k+s}^{k}y^{i-k}e^{T}\left(i\right)\Upsilon_{1}e\left(i\right)-V_{3}\left(k\right)\\ \;\;\;\;\;\;\;\;\;\;\;\;\;\;\;\;\;\;\;\;\;\;\;\;\;\;\;\;\;\;\leq \left(\tau_{M}-\tau_{m}+1\right)e^{T}\left(k\right)\Upsilon_{1}e\left(k\right)-y^{-\tau_{M}}e^{T}\left(\Delta_{k}\right)\;\Upsilon_{1}e\left(\Delta_{k}\right)\;+\left(y^{-1}-1\right)V_{3}\left(k\right). \end{array}$$

(A8) $$\begin{array}{c} \Delta V_{4}\left(k\right)=V_{4}\left(k+1\right)-V_{4}\left(k\right)\\ \;\;\;\;\;\;\;\;\;\;\;\;\;\;\;\;\;\;\;\;\;\;\;\;\;\;\;\;\;=\sum_{℘=-\tau_{M}}^{-\tau_{m}-1}\sum_{j=℘}^{-1}\sum_{i=k+1+j}^{k}y^{i-k}\beta^{T}\left(i\right)\Upsilon_{2}\beta \left(i\right)\;-\sum_{℘=-\tau_{M}}^{-\tau_{m}-1}\sum_{j=℘}^{-1}\sum_{i=k+j}^{k-1}y^{i-k-1}\beta^{T}\left(i\right)\Upsilon_{2}\beta \left(i\right)\\ \;\;\;\;\;\;\;\;\;\;\;\;\;\;\;\;\;\;\;\;\;\;\;\;\;\;\;\;\;\;\leq \frac{\tau_{m}\left(\tau_{m}+1\right)}{2}\beta^{T}\left(k\right)\Upsilon_{2}\beta \left(k\right)\;-y^{-1}\sum_{j=-\tau_{M}}^{-\tau_{m}-1}\sum_{i=k+j}^{k-1}\beta^{T}\left(i\right)\Upsilon_{2}\beta \left(i\right)+\left(y^{-1}-1\right)V_{4}\left(k\right). \end{array}$$

With the help of Lemma 1, we obtain

(A9) $$\begin{array}{c} \;\;\;-y^{-1}\sum_{j=-\tau_{M}}^{-\tau_{m}-1}\sum_{i=k+j}^{k-1}\beta^{T}\left(i\right)\Upsilon_{2}\beta \left(i\right)\\ =-y^{-1}\left[\sum_{j=-\tau (k)}^{-\tau_{m}-1}\sum_{i=k+j}^{k-1}\beta^{T}\left(i\right)\Upsilon_{2}\beta \left(i\right)+\sum_{j=-\tau_{M}}^{-\tau (k)-1}\sum_{i=k+j}^{k-1}\beta^{T}\left(i\right)\Upsilon_{2}\beta \left(i\right)\right]\\ \leq -\frac{y^{-1}}{\tau_{M}-\tau_{m}}\left(2\xi_{1}^{T}\Upsilon_{2}\xi_{1}+4\xi_{2}^{T}\Upsilon_{2}\xi_{2}+2\xi_{3}^{T}\Upsilon_{2}\xi_{3}+4\xi_{4}^{T}\Upsilon_{2}\xi_{4}\right)\\ =-\frac{y^{-1}}{\tau_{M}-\tau_{m}}\xi^{T}\left(k\right){\tilde{\Upsilon }}_{2}\xi \left(k\right). \end{array}$$

where

${\tilde{\Upsilon }}_{2}=diag\left\{2\Upsilon_{2},4\Upsilon_{2},2\Upsilon_{2},4\Upsilon_{2}\right\}$,

$\xi^{T}=\left[\xi_{1}\left(k\right),\xi_{2}\left(k\right),\xi_{3}\left(k\right),\xi_{4}\left(k\right)\right]$, $\xi_{1}\left(k\right)=e\left(\Delta_{k}\right)-\kappa_{2}$,

$\xi_{2}\left(k\right)=e\left(\Delta_{k}\right)-4\kappa_{2}+3\kappa_{5}$, $\xi_{3}\left(k\right)=e\left(\Delta_{M}\right)-\kappa_{3}$,

$\xi_{4}\left(k\right)=e\left(\Delta_{M}\right)-4\kappa_{3}+3\kappa_{6}$.

Combined with (A9), $\Delta V_{4}\left(k\right)$ can be bounded as

(A10) $$\Delta V_{4}\left(k\right)\leq \frac{\tau_{m}\left(\tau_{m}+1\right)}{2}\beta^{T}\left(k\right)\Upsilon_{2}\beta \left(k\right)+\left(y^{-1}-1\right)V_{4}\left(k\right)-\frac{y^{-1}}{\tau_{M}-\tau_{m}}\xi^{T}\left(k\right){\tilde{\Upsilon }}_{2}\xi \left(k\right).$$

(A11) $$\begin{array}{c} \Delta V_{5}\left(k\right)=V_{5}\left(k+1\right)-V_{5}\left(k\right)\\ \;\;\;\;\;\;\;\;\;\;\;\;\;\;\;\;\;\;\;\;\;\;\;\;\;\;\;\;\;=\left(\tau_{M}-\tau_{m}\right)\left[\sum_{j=-\tau_{M}}^{-\tau_{m}-1}\sum_{i=k+1+j}^{k}y^{i-k}\beta^{T}\left(i\right)\Upsilon_{3}\beta \left(i\right)\right.\left.\;-\sum_{j=-\tau_{M}}^{-\tau_{m}-1}\sum_{i=k+j}^{k-1}y^{i-k-1}\beta^{T}\left(i\right)\Upsilon_{3}\beta \left(i\right)\right]\\ \;\;\;\;\;\;\;\;\;\;\;\;\;\;\;\;\;\;\;\;\;\;\;\;\;\;\;\;\;\;\;\;\;\;\;\;\;\;\;\;+\tau_{m}\left[\sum_{j=-\tau_{m}}^{-1}\sum_{i=k+1+j}^{k}y^{i-k}\beta^{T}\left(i\right)\Upsilon_{4}\beta \left(i\right)\right.-\left.\sum_{j=-\tau_{m}}^{-1}\sum_{i=k+j}^{k-1}y^{i-k-1}\beta^{T}\left(i\right)\Upsilon_{4}\beta \left(i\right)\right]\\ \;\;\;\;\;\;\;\;\;\;\;\;\;\;\;\;\;\;\;\;\;\;\;\;\;\;\;\;\;\;\;\;\leq \beta^{T}\left(k\right)\left[{\left(\tau_{M}-\tau_{m}\right)}^{2}\Upsilon_{3}+\tau_{m}^{2}\Upsilon_{4}\right]\beta \left(k\right)-\left(\tau_{M}-\tau_{m}\right)y^{-\tau_{m}-1}\sum_{i=\Delta_{M}}^{\Delta_{m}-1}\beta^{T}\left(i\right)\Upsilon_{3}\beta \left(i\right)\\ \;\;\;\;\;\;\;\;\;\;\;\;\;\;\;\;\;\;\;\;\;\;\;\;\;\;\;\;\;\;\;\;\;\;\;\;\;\;\;-\tau_{m}\sum_{i=\Delta_{m}}^{k-1}\beta^{T}\left(i\right)\Upsilon_{4}\beta \left(i\right)+\left(y^{-1}-1\right)V_{5}\left(k\right). \end{array}$$

From Lemma 2, the following inequality holds:

(A12) $$\begin{array}{c} \;-\left(\tau_{M}-\tau_{m}\right)y^{-\tau_{m}-1}\sum_{i=\Delta_{M}}^{\Delta_{m}-1}\beta^{T}\left(i\right)\Upsilon_{3}\beta \left(i\right)\\ =-\left(\tau_{M}-\tau_{m}\right)y^{\tau_{m}+1}\left[\sum_{i=\Delta_{M}}^{\Delta_{k}-1}\beta^{T}\left(i\right)\Upsilon_{3}\beta \left(i\right)\right.+\left.\sum_{i=\Delta_{k}}^{\Delta_{m}-1}\beta^{T}\left(i\right)\Upsilon_{3}\beta \left(i\right)\right]\\ \leq -y^{\tau_{m}+1}\frac{\tau_{M}-\tau_{m}}{\tau \left(k\right)-\tau_{m}}\left[\zeta_{1}^{T}\left(k\right)\Upsilon_{3}\zeta_{1}\left(k\right)+3\zeta_{2}^{T}\left(k\right)\Upsilon_{3}\zeta_{2}\left(k\right)\right.+\left.5\zeta_{3}^{T}\left(k\right)\Upsilon_{3}\zeta_{3}\left(k\right)\right]\\ \;\;\;\;\;\;\;-y^{\tau_{m}+1}\frac{\tau_{M}-\tau_{m}}{\tau_{M}-\tau \left(k\right)}\left[\zeta_{4}^{T}\left(k\right)\Upsilon_{3}\zeta_{4}\left(k\right)+3\zeta_{5}^{T}\left(k\right)\Upsilon_{3}\zeta_{5}\left(k\right)+5\zeta_{6}^{T}\left(k\right)\Upsilon_{3}\zeta_{6}\left(k\right)\right]\\ =-y^{\tau_{m}+1}\zeta^{T}\left(k\right)\left[\begin{array}{cc} {\tilde{\Upsilon }}_{3} & R\\ {}* & {\tilde{\Upsilon }}_{3} \end{array}\right]\zeta \left(k\right) \end{array}$$

where

${\tilde{\Upsilon }}_{3}=diag\left\{\Upsilon_{3},3\Upsilon_{3},5\Upsilon_{3}\right\}$,

$\zeta^{T}\left(k\right)=\left[\zeta_{1}^{T}\left(k\right),\zeta_{2}^{T}\left(k\right),\zeta_{3}^{T}\left(k\right),\zeta_{4}^{T}\left(k\right),\zeta_{5}^{T}\left(k\right),\zeta_{6}^{T}\left(k\right)\right]$,

$\zeta_{1}\left(k\right)=e\left(\Delta_{m}\right)-e\left(\Delta_{k}\right)$, $\zeta_{2}\left(k\right)=e\left(\Delta_{m}\right)-e\left(\Delta_{k}\right)-2\kappa_{2}$,

$\zeta_{3}\left(k\right)=e\left(\Delta_{m}\right)-e\left(\Delta_{k}\right)+6\kappa_{2}-6\kappa_{5}$, $\zeta_{4}\left(k\right)=e\left(\Delta_{k}\right)-e\left(\Delta_{M}\right)$,

$\zeta_{5}\left(k\right)=e\left(\Delta_{k}\right)-e\left(\Delta_{M}\right)-2\kappa_{3}$, $\zeta_{6}\left(k\right)=e\left(\Delta_{k}\right)-e\left(\Delta_{M}\right)+6\kappa_{3}-6\kappa_{6}$.

Relying on Lemma 1, we can find that

(A13) $$-\tau_{m}\sum_{i=\Delta_{m}}^{k-1}\beta^{T}\left(i\right)\Upsilon_{4}\beta \left(i\right)\leq -\rho^{T}\left(k\right){\tilde{\Upsilon }}_{4}\rho \left(k\right)$$

where

${\tilde{\Upsilon }}_{4}=diag\left\{\Upsilon_{4},3z_{1}^{\tau_{m}}\Upsilon_{4},5z_{2}^{\tau_{m}}\Upsilon_{4}\right\},$$z_{1}^{\tau_{m}}=\frac{\tau_{m}+1}{\tau_{m}-1}$, $z_{2}^{\tau_{m}}=\frac{\left(\tau_{m}+1\right){\left(\tau_{m}+2\right)}^{2}}{\left(\tau_{m}-1\right)\left(\tau_{m}^{2}+11\right)}$,

$\rho^{T}\left(k\right)=\left[\rho_{1}^{T}\left(k\right),\rho_{2}^{T}\left(k\right),\rho_{3}^{T}\left(k\right)\right]$, $\rho_{1}\left(k\right)=e\left(k\right)-e\left(\Delta_{m}\right)$,

$\rho_{2}\left(k\right)=e\left(k\right)+e\left(\Delta_{m}\right)-2\kappa$, $\rho_{3}\left(k\right)=e\left(k\right)-e\left(\Delta_{m}\right)+6\kappa_{1}-6\kappa_{4}$.

Substituting (A12) and (A13) into (A11), one has

(A14) $$\begin{array}{c} \Delta V_{5}\left(k\right)\leq \beta^{T}\left(k\right)\left[{\left(\tau_{M}-\tau_{m}\right)}^{2}\Upsilon_{3}+\tau_{m}^{2}\Upsilon_{4}\right]\beta \left(k\right)+\left(y^{-1}-1\right)V_{5}\left(k\right)\\ \;\;\;\;\;\;\;\;\;\;\;\;\;\;\;\;\;\;\;\;\;\;\;-y^{\tau_{m}+1}\zeta^{T}\left(k\right)\left[\begin{array}{cc} {\tilde{\Upsilon }}_{3} & R\\ {}* & {\tilde{\Upsilon }}_{3} \end{array}\right]\zeta \left(k\right)\;-\rho^{T}\left(k\right){\tilde{\Upsilon }}_{4}\rho \left(k\right). \end{array}$$

According to Assumption 1 and (20),we can obtain following inequalities for $\hbar_{1},\hbar_{2}>0$

(A15) $$\hbar_{1}{\left[\begin{array}{c} e(k)\\ F(k) \end{array}\right]}^{T}\left[\begin{array}{cc} A_{1} & A_{2}\\ {}* & I_{nN} \end{array}\right]\left[\begin{array}{c} e(k)\\ F(k) \end{array}\right]\leq 0\;,$$

(A16) $$\hbar_{2}{\left[\begin{array}{c} e(\Delta_{k})\\ H(\Delta_{k}) \end{array}\right]}^{T}\left[\begin{array}{cc} M_{1} & M_{2}\\ {}* & I_{nN} \end{array}\right]\left[\begin{array}{c} e(\Delta_{k})\\ H(\Delta_{k}) \end{array}\right]\leq 0\;.$$

where

$A=\left[\begin{array}{cc} A_{1} & A_{2}\\ {}* & I_{nN} \end{array}\right],$$M=\left[\begin{array}{cc} M_{1} & M_{2}\\ {}* & I_{nN} \end{array}\right]$.

$A_{1}=\frac{{\tilde{A}}_{1}^{T}{\tilde{A}}_{2}+{{\tilde{A}}_{2}}^{T}{\tilde{A}}_{1}}{2}$, $A_{2}=-\frac{{{\tilde{A}}_{1}}^{T}+{{\tilde{A}}_{2}}^{T}}{2}$, $M_{1}=\frac{{{\tilde{M}}_{1}}^{T}{\tilde{M}}_{2}+{{\tilde{M}}_{2}}^{T}{\tilde{M}}_{1}}{2}$, $M_{2}=-\frac{{{\tilde{M}}_{1}}^{T}+{{\tilde{M}}_{2}}^{T}}{2}$,

For symmetric matrix Q>0, it follows from (11) that

(A17) $$\begin{array}{c} 0=2e^{T}\left(k\right)Q\left[e(k+1)-e(k+1)\right]\\ \;\;\;\;\;\;\;=2e^{T}\left(k\right)Q\left\{\sum_{l=1}^{r}\eta_{l}\left(\theta \left(k\right)\right)\left[A_{l}e\left(k\right)+B_{l1}F\left(k\right)+B_{l2}H\left(\Delta_{k}\right)\right.\right.\\ \left.\;\;\;\;\;\;\;\;\;\;\;\;\;\;+\left.c\left(G_{l}⊗\Gamma \right)e\left(\Delta_{k}\right)+w\left(k\right)-K_{l}\varepsilon \left(k\right)-K_{l}e\left(k\right)\right]-e\left(k+1\right)\right\}\\ \;\;\;\;\;\;\;=2e^{T}\left(k\right)\sum_{l=1}^{r}\eta_{l}\left(\theta \left(k\right)\right)\left[QA_{l}e\left(k\right)+QB_{l1}F\left(k\right)+QB_{l2}H\left(\Delta_{k}\right)\right.\\ \;\;\;\;\;\;\;\;\;\;\;\;\;\;+\left.cQ\left(G_{l}⊗\Gamma \right)e\left(\Delta_{k}\right)+Qw\left(k\right)-K_{l}\varepsilon \left(k\right)-K_{l}e\left(k\right)\right]-2e^{T}\left(k\right)Qe\left(k+1\right) \end{array}$$

Repeating the process from (A1) to (A17), we obtain

(A18) $$\begin{array}{c} \;\;\;\;\;\;\;\;\Delta V\left(k\right)-\left(y^{-1}-1\right)V\left(k\right)-w{\left(k\right)}^{T}\Upsilon_{5}w\left(k\right)\\ =\beta \left(k\right)Q\;\beta \left(k\right)+2e^{T}\left(k\right)Qe\left(k+1\right)-\left(1+y^{-1}\right)e^{T}\left(k\right)Qe\left(k\right)\\ \;\;\;\;\;\;+\sum_{i-1}^{N}\sigma_{i}\left[\left(\frac{y}{\;{ƛ}_{i}}-1\right)d_{i}\left(k\right)+yE_{i}^{T}{\tilde{\Omega }}_{i}E_{i}\right]+\left(\tau_{M}-\tau_{m}+1\right)e^{T}\left(k\right)\Upsilon_{1}e\left(k\right)\\ \;\;\;\;\;\;-y^{-\tau_{M}}e^{T}\left(\Delta_{k}\right)\;\Upsilon_{1}e\left(\Delta_{k}\right)+\frac{\tau_{m}\left(\tau_{m}+1\right)}{2}\beta^{T}\left(k\right)\Upsilon_{2}\beta \left(k\right)\;\;\\ \;\;\;\;\;\;-\frac{y^{-1}}{\tau_{M}-\tau_{m}}\xi^{T}\left(k\right){\tilde{\Upsilon }}_{2}\xi \left(k\right)+\beta^{T}\left(k\right)\left[{\left(\tau_{M}-\tau_{m}\right)}^{2}\Upsilon_{3}+\tau_{m}^{2}\Upsilon_{4}\right]\beta \left(k\right)\\ \;\;\;\;\;\;\;-y^{\tau_{m}+1}\zeta^{T}\left(k\right)\left[\begin{array}{cc} {\tilde{\Upsilon }}_{3} & R\\ {}* & {\tilde{\Upsilon }}_{3} \end{array}\right]\zeta \left(k\right)\;-\rho^{T}\left(k\right){\tilde{\Upsilon }}_{4}\rho \left(k\right)-w{\left(k\right)}^{T}\Upsilon_{5}w\left(k\right)\;\\ \;\;\;\;\;\;\;\;+\hbar^{*}\sum_{i=1}^{N}\sigma_{i}\left[d_{i}\left(k\right)+E_{i}^{T}\Omega_{i}E_{i}\right]+\mathrm{Sym}\left\{\sum_{l=1}^{r}\eta_{l}\left(\theta \left(k\right)\right)\left[e^{T}\left(k\right)QA_{l}e\left(k\right)\right.\right.\\ \;\;\;\;\;\;\;\;+e^{T}\left(k\right)QB_{l1}F\left(k\right)+e^{T}\left(k\right)QB_{l2}H\left(\Delta_{k}\right)+ce^{T}\left(k\right)Q\left(G_{l}⊗\Gamma \right)e\left(\Delta_{k}\right)\\ \;\;\;\;\;\;\left.\left.\;\;+e^{T}\left(k\right)Qw\left(k\right)-e^{T}\left(k\right)K_{l}\varepsilon \left(k\right)-e^{T}\left(k\right)K_{l}e\left(k\right)\right]\right\}\\ \;\;\;\;\;\;\;\;-2e^{T}\left(k\right)Qe\left(k+1\right)-\hbar_{1}{\left[\begin{array}{c} e(k)\\ F(k) \end{array}\right]}^{T}\left[\begin{array}{cc} A_{1} & A_{2}\\ {}* & I_{N} \end{array}\right]\left[\begin{array}{c} e(k)\\ F(k) \end{array}\right]\\ \;\;\;\;\;\;\;\;-\hbar_{2}{\left[\begin{array}{c} e(\Delta_{k})\\ H(\Delta_{k}) \end{array}\right]}^{T}\left[\begin{array}{cc} M_{1} & M_{2}\\ {}* & I_{N} \end{array}\right]\left[\begin{array}{c} e(\Delta_{k})\\ H(\Delta_{k}) \end{array}\right]\\ \leq \sum_{l=1}^{r}\eta_{l}\left(\theta \left(k\right)\right)\left[\gamma^{T}\left(k\right)(\Psi_{1}+\Psi_{2}^{T}\Theta \Psi_{2}\right.\;\left.)\gamma (k)\right]+\sum_{i=1}^{N}\frac{1}{\;{ƛ}_{i}}\left(y\sigma_{i}+\hbar^{*}\right) \end{array}$$

where $\Psi_{1}$, $\Psi_{2}$, and Θ are defined in (20).

Then by the Schur complement theory, it is no difficult to get the following inequality from (20):

(A19) $$\Psi_{1}+\Psi_{2}^{T}\Theta \Psi_{2}<0.$$

Thus

(A20) $$\Delta V\left(k\right)\leq \left(y^{-1}-1\right)V\left(k\right)+w^{T}\left(k\right)\Upsilon_{5}w\left(k\right)+L.$$

where $L=\sum_{i=1}^{N}\frac{1}{\;{ƛ}_{i}}\left(y\sigma_{i}+\hbar^{*}\right)$

Based on $V\left(k\right)<y^{-1}V\left(k-1\right)<\cdots <y^{-T_{m}}V\left(0\right)$ from the result in [25], V(k) can be derived as

(A21) $$\begin{array}{c} V\left(k\right)\leq y^{-1}V_{q}\left(k-1\right)+w^{T}\left(k-1\right)\Upsilon_{5}w\left(k-1\right)+L\\ \;\;\;\;\;\;\;\;\;\;\;\;\;\;\;\;\;\;\;\;\leq y^{-2}V_{q}\left(k-2\right)+y^{-1}w^{T}\left(k-1\right)\Upsilon_{5}w\left(k-1\right)\\ \;\;\;\;\;\;\;\;\;\;\;\;\;\;\;\;\;\;\;\;\;\;\;\;\;\;+w^{T}\left(k-2\right)\Upsilon_{5}w\left(k-2\right)+y^{-1}L+L\\ \;\;\;\;\;\;\;\;\;\;\;\;\;\;\;\;\;\;\;\;\;\vdots \\ \;\;\;\;\;\;\;\;\;\;\;\;\;\;\;\;\;\;\;\;\leq y^{-T_{m}}V\left(0\right)+\bar{w}\sum_{i=0}^{T_{m}-1}y^{-T_{m}+i+1}w^{T}\left(i\right)w\left(i\right)+\frac{1-y^{-T_{m}}}{1-y^{-1}}L\\ \;\;\;\;\;\;\;\;\;\;\;\;\;\;\;\;\;\;\;\;\leq y^{-T_{m}}V\left(0\right)+y^{-T_{m}}\tilde{w}\bar{w}+\frac{1}{1-y^{-1}}L. \end{array}$$

By means of Lemma 3 and (20), the initial value of V(k) is denoted as

(A22) $$\begin{array}{c} V\left(0\right)=e^{T}\left(0\right)Qe\left(0\right)+\sum_{i=1}^{N}y\sigma_{i}d_{i}\left(0\right)+\sum_{i=-\tau (0)}^{-1}y^{i+1}e^{T}\left(i\right)\Upsilon_{1}e\left(i\right)\\ \;\;\;\;\;\;\;\;\;\;\;\;\;\;\;\;\;\;\;\;\;\;\;\;+\sum_{j=-\tau_{M}}^{-1}\sum_{℘=j}^{-1}\sum_{i=℘}^{-1}y^{i+1}\beta^{T}\left(i\right)\Upsilon_{2}\beta \left(i\right)+\left(\tau_{M}-\tau_{m}\right)\sum_{j=-\tau_{M}}^{-\tau_{m}-1}\sum_{i=j}^{-1}y^{i+1}\beta^{T}\left(i\right)\Upsilon_{3}\beta \left(i\right)\\ \;\;\;\;\;\;\;\;\;\;\;\;\;\;\;\;\;\;\;\;\;\;\;\;\;\;\;\;+\tau_{m}\sum_{j=-\tau_{m}}^{-1}\sum_{i=j}^{-1}y^{i+1}\beta^{T}\left(i\right)\Upsilon_{4}\beta \left(i\right)\\ \;\;\;\;\;\;\;\;\;\;\;\;\;\;\;\;\;\;\;\;\;\;\;\leq m_{1}\lambda_{1}+y\sum_{i=1}^{N}\sigma_{i}d_{i0}+m_{1}\lambda_{2}\sum_{i=-\tau_{M}}^{-1}y^{i+1}+\varpi \lambda_{3}\sum_{j=-\tau_{M}}^{-\tau_{m}-1}\sum_{℘=j}^{-1}\sum_{i=℘}^{-1}y^{i+1}\\ \;\;\;\;\;\;\;\;\;\;\;\;\;\;\;\;\;\;\;\;\;\;\;\;\;\;\;\;+\left(\tau_{M}-\tau_{m}\right)\varpi \lambda_{4}\sum_{j=-\tau_{M}}^{-\tau_{m}-1}\sum_{i=j}^{-1}y^{i+1}\;+\tau_{m}\varpi \lambda_{5}\sum_{j=-\tau_{m}}^{-1}\sum_{i=j}^{-1}y^{i+1}\\ \;\;\;\;\;\;\;\;\;\;\;\;\;\;\;\;\;\;\;\;\;\;\;\;=m_{1}\left[\lambda_{1}+o_{1}\lambda_{2}\right]+\varpi \left[o_{2}\lambda_{3}\right.+\left(\tau_{M}-\tau_{m}\right)o_{3}\lambda_{4}+\left.\tau_{m}o_{4}\lambda_{5}\right]+y\sum_{i=1}^{N}\sigma_{i}d_{i0}.\\ \;\;\;\;\;\;\;\;\;\;\;\;\;\;\;\;\;\;\;\;\;\;\;\;\;=m_{1}L_{1}+\varpi L_{2}+y\sum_{i=1}^{N}\sigma_{i}d_{i0}. \end{array}$$

The combination (A20) and (A21) can obtain

(A23) $$V\left(k\right)<y^{-T_{m}}\left[m_{1}L_{1}+\varpi L_{2}+y\sum_{i=1}^{N}\sigma_{i}d_{i0}+\tilde{w}\bar{w}\right].$$

Recalling (20) results in

(A24) $$V\left(k\right)⩾\lambda_{0}e^{T}\left(k\right)\Phi e\left(k\right)$$

Namely, we further get the following inequality

(A25) $$e^{T}\left(k\right)\Phi e\left(k\right)<\frac{y^{-T_{m}}\left[m_{1}L_{1}+\varpi L_{2}+y\sum_{i=1}^{N}\sigma_{i}d_{i0}+\tilde{w}\bar{w}\right]}{\lambda_{0}}\leq m_{2}.$$

According to the given condition in Definition 1 and the bound of V(k) in (A21), we deduce that

(A26) $$\lambda_{0}m_{2}\leq y^{-T_{m}}V\left(0\right)+y^{-T_{m}}\tilde{w}\bar{w}+\frac{1}{1-y^{-1}}L$$

From (20), L should satisfy $L\leq m_{2}\left(1-y^{-1}\right)$, so (A26) is further derived as

(A27) $$\left(\lambda_{0}-1\right)m_{2}\leq y^{-T_{m}}\left(V\left(0\right)+\tilde{w}\bar{w}\right)$$

Then, the upper bound of finite time $T_{m}$ is described by

minTT⩾logy−1(λ0−1)m2(λ0−1)m2V(0)+w˜w¯V(0)+w˜w¯,T∈Z

when m2<V(0)+w˜w¯V(0)+w˜w¯(λ0−1)(λ0−1) holds, otherwise $T_{m}=0$.

As a result, the closed-loop TSFDCNs can reach synchronization in finite time $T_{m}$ with respect to $\left(m_{1},m_{2},\Phi ,\tilde{w},T_{m}\right)$. The proof of Theorem 1 is accomplished.

## Appendix B. Proof of Theorem 2

Define Lyapunov-Krasovskii functional candidate as

(A28) $$\begin{array}{c} V\left(k\right)=e^{T}\left(k\right)Qe\left(k\right)+\sum_{i=k-\tau }^{k-1}y^{i-k+1}e^{T}\left(i\right)\Upsilon_{1}e\left(i\right)+\sum_{j=-\tau }^{-1}\sum_{j=k+j}^{k-1}y^{i-k+1}\beta^{T}\left(i\right)\Upsilon_{2}\beta \left(i\right)\\ \;\;\;\;\;\;\;\;\;\;\;\;\;\;\;\;\;\;\;\;\;\;\;\;\;\;\;\;\;+\sum_{j=-\tau }^{-1}\sum_{℘=j}^{-1}\sum_{j=k+℘}^{k-1}y^{i-k+1}\beta^{T}\left(i\right)\Upsilon_{3}\beta \left(i\right)+\sum_{i=1}^{N}y\sigma_{i}d_{i}\left(k\right)\;, \end{array}$$

let

(A29) $$\begin{array}{cc} \; & {\tilde{\gamma }}^{T}\left(k\right)=\left[e^{T}\left(k\right),e^{T}\left(\Delta_{\tau }\right),{\tilde{\kappa }}_{1}^{T},{\tilde{\kappa }}_{2}^{T},\right.F^{T}\left(k\right),H^{T}\left(k\right),\left.d^{1/2}\left(k\right),\varepsilon^{T}\left(k\right)\right],\\ \; & {\tilde{\kappa }}_{1}=\frac{1}{\tau +1}\sum_{i=\Delta_{\tau }}^{k}e(i),{\tilde{\kappa }}_{2}=\frac{2}{\left(\tau +1)(\tau +2\right)}\sum_{j=-\tau }^{0}\sum_{i=k+j}^{k}e(i). \end{array}$$

The forward difference of V(k) is calculated as

(A30) $$\begin{array}{c} \Delta V\left(k\right)\leq \beta \left(k\right)Q\;\beta \left(k\right)+2e^{T}\left(k\right)Qe\left(k+1\right)\\ \;\;\;\;\;\;\;\;\;\;\;\;\;\;\;\;\;\;\;\;\;\;\;\;\;\;\;\;\;\;\;\;\;\;-\left(1+y^{-1}\right)e^{T}\left(k\right)Qe\left(k\right)+e^{T}\left(k\right)\Upsilon_{1}e\left(k\right)\\ \;\;\;\;\;\;\;\;\;\;\;\;\;\;\;\;\;\;\;\;\;\;\;\;\;\;\;\;\;\;\;\;\;\;-y^{-\tau }e^{T}\left(\Delta_{\tau }\right)\Upsilon_{1}e\left(\Delta_{\tau }\right)\;+\tau^{2}\beta^{T}\left(k\right)\Upsilon_{2}\beta \left(k\right)\\ \;\;\;\;\;\;\;\;\;\;\;\;\;\;\;\;\;\;\;\;\;\;\;\;\;\;\;\;\;\;\;\;\;-\tau \sum_{i=\Delta_{\tau }}^{k-1}\beta^{T}\left(i\right)\Upsilon_{2}\beta \left(i\right)+\frac{\tau (\tau +1)}{2}\beta^{T}\left(k\right)\Upsilon_{3}\beta \left(k\right)\\ \;\;\;\;\;\;\;\;\;\;\;\;\;\;\;\;\;\;\;\;\;\;\;-y^{-\tau }\sum_{j=-\tau }^{-1}\sum_{i=j+k}^{k-1}\beta^{T}\left(i\right)\Upsilon_{3}\beta \left(i\right)+\left(y^{-1}-1\right)V\left(k\right)\\ \;\;\;\;\;\;\;\;\;\;\;\;\;\;\;\;\;\;\;\;\;\;\;\;\;\;\;\;\;\;\;\;\;\;\;+\sum_{i-1}^{N}\sigma_{i}\left[\left(\frac{y}{\;{ƛ}_{i}}-1\right)d_{i}\left(k\right)+yE_{i}^{T}{\tilde{\Omega }}_{i}E_{i}\right]. \end{array}$$

According to Lemma 1, we have

(A31) $$-\tau \sum_{i=\Delta_{\tau }}^{k-1}\beta^{T}\left(i\right){\tilde{\Upsilon }}_{2}^{\tau }\beta \left(i\right)\leq -{\tilde{\gamma }}^{T}\left(k\right){\tilde{\Lambda }}_{1}^{T}{\tilde{\Upsilon }}_{2}^{\tau }{\tilde{\Lambda }}_{1}\tilde{\gamma }\left(k\right)$$

and similarly,

(A32) $$-y^{-\tau }\sum_{j=-\tau }^{-1}\sum_{i=j+k}^{k-1}\beta^{T}\left(i\right)\Upsilon_{3}\beta \left(i\right)\leq -y^{-\tau }\frac{2(\tau +1)}{\tau }{\tilde{\gamma }}^{T}\left(k\right){\tilde{\Lambda }}_{2}^{T}{\tilde{\Upsilon }}_{3}{\tilde{\Lambda }}_{2}\tilde{\gamma }\left(k\right).$$

where ${\tilde{\Upsilon }}_{2}=diag\left\{\Upsilon_{2},3z_{1}\left(\tau \right)\Upsilon_{2},5z_{2}\left(\tau \right)\Upsilon_{2}\right\}$, ${\tilde{\Upsilon }}_{3}=diag\left\{\Upsilon_{3},3z_{2}\left(\tau \right)\Upsilon_{3}\right\}$,

$z_{1}\left(\tau \right)=\frac{\tau +1}{\tau -1}$, $z_{2}\left(\tau \right)=\frac{\left(\tau +1\right){\left(\tau +2\right)}^{2}}{\left(\tau -1\right)\left(\tau^{2}+11\right)}$, $z_{3}\left(\tau \right)=\frac{\tau +2}{\tau -1}$,

${\tilde{\Lambda }}_{1}={\left(e\left(k\right)-e\left(\Delta_{\tau }\right),e\left(k\right)+e\left(\Delta_{\tau }\right)-2{\tilde{\kappa }}_{1},e\left(k\right)-e\left(\Delta_{\tau }\right)+6{\tilde{\kappa }}_{1}-6{\tilde{\kappa }}_{2}\right)}^{T}$,

${\tilde{\Lambda }}_{2}={\left(e\left(\Delta_{\tau }\right)-{\tilde{\kappa }}_{1},e\left(\Delta_{\tau }\right)-4{\tilde{\kappa }}_{1}+3{\tilde{\kappa }}_{2}\right)}^{T}$.

By taking (A6) and (A15)–(A17) in Theorem 1 into account, we obtain

(A33) $$\Delta V\left(k\right)-\left(y^{-1}-1\right)V\left(k\right)\leq {\tilde{\gamma }}^{T}\left(k\right)\left({\tilde{\Psi }}_{1}+{\tilde{\Psi }}_{2}^{T}\tilde{\Theta }{\tilde{\Psi }}_{2}\right)\tilde{\gamma }\left(k\right)\;+\sum_{i=1}^{N}\frac{1}{\;{ƛ}_{i}}\left(y\sigma_{i}+\hbar^{*}\right)$$

where ${\tilde{\Psi }}_{1}+{\tilde{\Psi }}_{2}^{T}\tilde{\Theta }{\tilde{\Psi }}_{2}<0$ based on Schur complement theory. As similar with (A21), we notice that V(k)<y−NV(0)+LL1−y−1(1−y−1).Then the initial value of V(k) is described as

(A34) $$\begin{array}{c} V\left(0\right)=e^{T}\left(0\right)Qe\left(0\right)+\sum_{i=-\tau }^{-1}y^{i+1}e^{T}\left(i\right)\Upsilon_{1}e\left(i\right)+\sum_{j=-\tau }^{-1}\sum_{j=j}^{-1}y^{i+1}\beta^{T}\left(i\right)\Upsilon_{2}\beta \left(i\right)\\ \;\;\;\;\;\;\;\;\;\;\;\;\;\;\;\;\;\;\;\;\;\;\;\;\;+\sum_{j=-\tau }^{-1}\sum_{℘=j}^{-1}\sum_{j=℘}^{-1}y^{i+1}\beta^{T}\left(i\right)\Upsilon_{3}\beta \left(i\right)+\sum_{i=1}^{N}y\sigma_{i}d_{i}\left(0\right)\\ \;\;\;\;\;\;\;\;\;\;\;\;\;\;\;\;\;\;\;\;\;=m_{1}\lambda_{1}+m_{1}\lambda_{2}\sum_{i=-\tau }^{-1}y^{i+1}+\varpi \lambda_{3}\sum_{j=-\tau }^{-1}\sum_{i=j}^{-1}y^{i+1}+\varpi \lambda_{4}\sum_{j=-\tau }^{-1}\sum_{℘=j}^{-1}\sum_{i=℘}^{-1}y^{i+1}\\ \;\;\;\;\;\;\;\;\;\;\;\;\;\;\;\;\;\;\;\;\;\;\;\;\;\;+y\sum_{i=1}^{N}\sigma_{i}d_{i0}\\ \;\;\;\;\;\;\;\;\;\;\;\;\;\;\;\;\;\;\;\;\;\;=m_{1}{\tilde{L}}_{1}+\varpi {\tilde{L}}_{2}+y\sum_{i=1}^{N}\sigma_{i}d_{i0}. \end{array}$$

Based on Lemma 3, we get $V\left(k\right)⩾\lambda_{0}e^{T}\left(k\right)\Phi e\left(k\right)$ from (24). It is concluded that

(A35) $$e^{T}\left(k\right)\Phi e\left(k\right)<\frac{y^{-T_{m}}\left(m_{1}{\tilde{L}}_{1}+\varpi {\tilde{L}}_{2}+y\sum_{i=1}^{N}\sigma_{i}d_{i0}\right)}{\lambda_{0}}\leq m_{2}.$$

Consider the process in Theorem 1, we can further calculate the maximum finite time $T_{m}$ of synchronization as minTT⩾logy−1(λ0−1)m2(λ0−1)m2V(0)V(0),T∈Z for m2<V(0)V(0)(λ0−1)(λ0−1). So the finite-time synchronization of DCNs is realized with respect to $\left(m_{1},m_{2},\Phi ,T_{m}\right)$. The proof is accomplished.
